# Design and Characterization of a Multistage Peptide-Based Vaccine Platform to Target *Mycobacterium tuberculosis* Infection

**DOI:** 10.1021/acs.bioconjchem.3c00273

**Published:** 2023-08-22

**Authors:** Chiara Bellini, Emil Vergara, Fruzsina Bencs, Kinga Fodor, Szilvia Bősze, Denis Krivić, Bernadett Bacsa, Sára Eszter Surguta, József Tóvári, Rajko Reljic, Kata Horváti

**Affiliations:** † MTA-TTK Lendület “Momentum” Peptide-Based Vaccines Research Group, Institute of Materials and Environmental Chemistry, Research Centre for Natural Sciences, Budapest 1117, Hungary; ‡ Hevesy György PhD School of Chemistry, Eötvös Loránd University, Budapest 1117, Hungary; § Institute for Infection and Immunity, St. George’s, University of London, London SW17 0RE, U.K.; ∥ Laboratory of Structural Chemistry and Biology, Institute of Chemistry, Eötvös Loránd University, Budapest 1117, Hungary; ⊥ Department of Laboratory Animal Science and Animal Protection, University of Veterinary Medicine, Budapest 1078, Hungary; # ELKH-ELTE Research Group of Peptide Chemistry, Eötvös Loránd Research Network (ELKH), Eötvös Loránd University, Budapest 1117, Hungary; ∇ Division of Medical Physics and Biophysics, Gottfried Schatz Research Center, Medical University of Graz, 8010 Graz, Austria; ○ Department of Experimental Pharmacology and National Tumor Biology Laboratory, National Institute of Oncology, Budapest 1122, Hungary

## Abstract

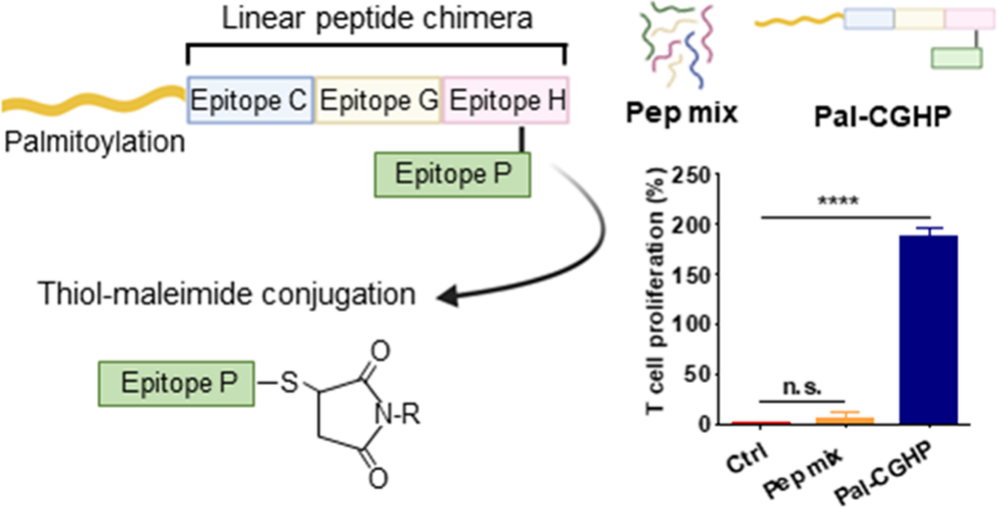

The complex immunopathology of*Mycobacterium tuberculosis*(*Mtb*) is one of the main challenges in developing a novel vaccine against this pathogen, particularly regarding eliciting protection against both active and latent stages. Multistage vaccines, which contain antigens expressed in both phases, represent a promising strategy for addressing this issue, as testified by the tuberculosis vaccine clinical pipeline. Given this approach, we designed and characterized a multistage peptide-based vaccine platform containing CD4+ and CD8+ T cell epitopes previously validated for inducing a relevant T cell response against *Mtb*. After preliminary screening, CFP10 (32–39), GlfT2 (4–12), HBHA (185–194), and PPE15 (1–15) were selected as promising candidates, and we proved that the **PM1** pool of these peptides triggered a T cell response in *Mtb*-sensitized human peripheral blood mononuclear cells (PBMCs). Taking advantage of the use of thiol-maleimide chemoselective ligation, we synthesized a multiepitope conjugate (**Ac-CGHP**). Our results showed a structure–activity relationship between the conjugation and a higher tendency to fold and assume an ordered secondary structure. Moreover, the palmitoylated conjugate (**Pal-CGHP**) comprising the same peptide antigens was associated with an enhanced cellular uptake in human and murine antigen-presenting cells and a better immunogenicity profile. Immunization study, conducted in BALB/c mice, showed that **Pal-CGHP** induced a significantly higher T cell proliferation and production of IFNγ and TNFα over **PM1** formulated in the Sigma Adjuvant System.

## Introduction

Tuberculosis (TB) remains an urgent global health priority, especially after the COVID-19 pandemic severely impacted the efforts to eradicate it.^1^ Developing a more effective vaccine is pivotal in containing the epidemic. Bacille Calmette-Guérin (BCG), the only available vaccine, provides inadequate and inconsistent protection against pulmonary TB in adults, the primary source of disease transmission.^2,3^ Nevertheless, progress in research toward a better vaccine is challenging because of the complex immunopathology of *Mycobacterium tuberculosis(Mtb)* and the lack of immune correlates of protection.

Moreover, *Mtb* can survive inside infected phagocytes through immune-suppressing mechanisms, such as inhibiting phagosome maturation and downregulating antigen presentation.^4,5^ The bacteria’s persistence triggers the recruitment of adaptive immune cells and the formation of granulomas. As *Mtb* enters the dormant stage, it changes the expression of its antigen repertoire and establishes an immunological equilibrium with the host.^6^

In this regard, the development of multistage TB vaccines, such as M72/AS01E, H56:IC31, and ID93/GLA-SE, has been driven by the need to protect against the active and latent stage of infection.^7^

Peptide-based multiepitope vaccines can incorporate MHC class I and II epitopes to broaden the T cell response. In addition, they are easier and faster to manufacture over protein-based subunit vaccines. Peptide-based multiepitope vaccines have shown promising results against some tumors and infectious agents but have so far only advanced to the preclinical stage in TB vaccine research.^8−10^ Nonetheless, the design of this vaccine type requires careful epitope selection and a proper formulation to overcome their inherent challenges of poor immunogenicity and *in vivo* stability.

In this work, we selected epitopes from protein antigens associated with various activities of the *Mtb* life cycle, including pathogenicity, metabolism, and cell wall maintenance (Table 1). Since it is crucial to induce a comprehensive immune response when targeting complex pathogens, such as *Mtb*, the chosen epitopes have been formerly validated for inducing a T cell-mediated response in active and latent infection.^11−35^

**Table 1:** List of Selected CD4+ and CD8+ T Cell Epitopes

	active stage peptide antigens				
gene name	protein name^a^	protein function	epitope name	MHC restriction	refs
***Rv3908c***	galactofuranosyltransferase GlfT2	cell wall polysaccharide biosynthesis	GlfT2 (4–12)	I	(11, 12)
***Rv1886c***	diacylglycerol acyltransferase/mycolyltransferase Ag85B	cell wall mycoloylation, fibronectin binder	Ag85B (41–48)	I	(11, 13)
***Rv1384***	carbamoyl-phosphate synthase large chain	pyrimidine metabolism	CarB (744–754)	I	(11)
***Rv1436***	glyceraldehyde-3-phosphate dehydrogenase	glycolytic enzyme	gap (112–122)	I	(11, 14)
***Rv3874***	ESAT-6-like protein EsxB	virulence-associated type VII secretion system	CFP10 (32–39)	I	(15, 16)
			CFP10 (11–25)	II	
***Rv0288***	ESAT-6-like protein EsxH	virulence-associated type VII secretion system	TB10.4 (20–28)	I	(17, 18)
***Rv0867c***	resuscitation-promoting factor RpfA	peptidoglycan-hydrolyzing enzyme	RpfA (377–391)	II	(19, 21)
***Rv1174c***	β sliding clamp	unknown function	TB8.4 (69–83)	II	(20, 21)
***Rv1334***	CysO-cysteine peptidase	cysteine synthesis	mec (2–20)	II	(22, 23)

	latent stage peptide antigens				
gene name	protein name^a^	protein function	epitope name	MHC restriction	refs
***Rv0475***	heparin-binding hemagglutinin	extrapulmonary dissemination	HBHA (185–194)	II	(11, 24)
***Rv0440***	60 kDa chaperonin 2	protein folding	Grol2 (63–78)	II	(11, 32)
***Rv0125***	probable serine protease PepA	probable serine protease	Mtb32a (309–318)	I	(25, 33)
***Rv1733c***	probable membrane protein Rv1733c	unknown function	Rv1733c (63–77)	II	(34, 35)
***Rv1039c***	PPE family protein PPE15	potential role in lipid metabolism	PPE15 (1–15)	II	(26−28)
***Rv0341***	isoniazid-induced protein IniB	unknown function, probably involved in isoniazid tolerance	IniB (33–45)	II	(29−31)

a Protein name was reported according to the Universal Protein Resource (UniProt) database.

A screening process for the peptide antigens panel was performed considering both synthetic aspects, like feasibility and peptide sequence, as well as biological relevance, such as the internalization efficiency in antigen-presenting cells (APCs) and their antigenic profile. Consequently, the most promising CD4+ and CD8+ T cell epitopes expressed in active and latent TB were chosen and incorporated into a branched multiepitope conjugate using solid-phase peptide synthesis (SPPS), fatty acid chain elongation, and chemoselective ligation.

## Results and Discussion

### Peptide Synthesis, Cytotoxicity, and Cellular Uptake Studies

The peptides were synthesized using the Fmoc/^t^Bu strategy, and the fluorescein-labeled derivative of each peptide was obtained by coupling 5(6)-carboxyfluorescein (5(6)-FAM) at the N*-*terminus to perform cellular uptake studies. The analytical characterization of the peptides is shown in Table 2 and Figures S1–S4, while analytical parameters of 5(6)-FAM derivatives are summarized in Table S1.

**Table 2:** Analytical Characterization of Synthesized Peptides

epitope name	sequence	*M*_mo_ calc.	*M*_mo_ meas.^a^	RT (min)^b^	GRAVY^c^
GlfT2 (4–12)	LAASLLSRV	927.5865	927.5848	13.1	1.456
Ag85B (41–48)	FSRPGLPV	870.5076	870.5036	12.2	0.237
CarB (744–754)	DEETLQGYITR	1322.6466	1322.6443	12.5	–1.209
gap (112–122)	KAKGHLDAGAK	1093.6356	1093.6341	9.1	–0.909
CFP10 (32–39)	VESTAGSL	761.3919	761.3907	10.1	0.450
CFP10 (11–25)	LAQEAGNFERISGDL	1617.8111	1617.8079	12.8	–0.340
TB10.4 (20–28)	GYAGTLQSL	907.4763	907.4741	12.9	0.256
RpfA (377–391)	AYTKKLWQAIRAQDV	1788.9999	1788.9967	12.8	–0.520
TB8.4 (69–83)	LRNFLAAPPPQRAANle	1632.9576	1632.9552	12.9	0.04
mec (2–20)	LLRKGTVYVLVIRADLVNA	2111.2943	2111.2899	16.0	0.937
HBHA (185–194)	KAPAKKAAAK	981.6447	981.6427	5.4	–0.820
Grol2 (63–78)	DPYEKIGAELVK	1359.7398	1359.7329	12.5	–0.608
Mtb32a (309–318)	GAPINSATAM	930.4593	930.4567	10.9	0.480
Rv1733c (63–77)	AGTAVQDSRSHVYAH	1596.7757	1596.7690	9.8	–0.540
PPE15 (1–15)	MDFGALPPEINSARM	1646.7909	1646.7840	13.5	–0.060
IniB (33–45)	GLIDIAPHQISSV	1347.7510	1347.7508	10.5	0.731

a *M*_mo_ Meas. (monoisotopic molecular mass) measured on a Thermo Scientific Q Exactive Focus Hybrid Quadrupole-Orbitrap Mass Spectrometer.

b Retention time on Phenomenex Jupiter C12, gradient: 5–100% B, 20 min. According to the high-performance liquid chromatography (HPLC) analysis, the purity of the peptides was always above 95%.

c GRAVY (grand average of hydropathicity) was calculated by Expasy, the Swiss Bioinformatics Resource Portal.^36^

In the rationale design of our vaccine platform, we assessed the ultimate primary structure’s implications. Indeed, the amino acid sequence influences the spatial conformation of the peptide backbone and also holds the potential to contribute to side reactions, such as aspartimide formation. Additionally, we calculated the hydropathicity of the peptides (GRAVY index) since the hydropathic character of the final compound can impact the behavior and interactions within biological systems.

**Figure 1:**
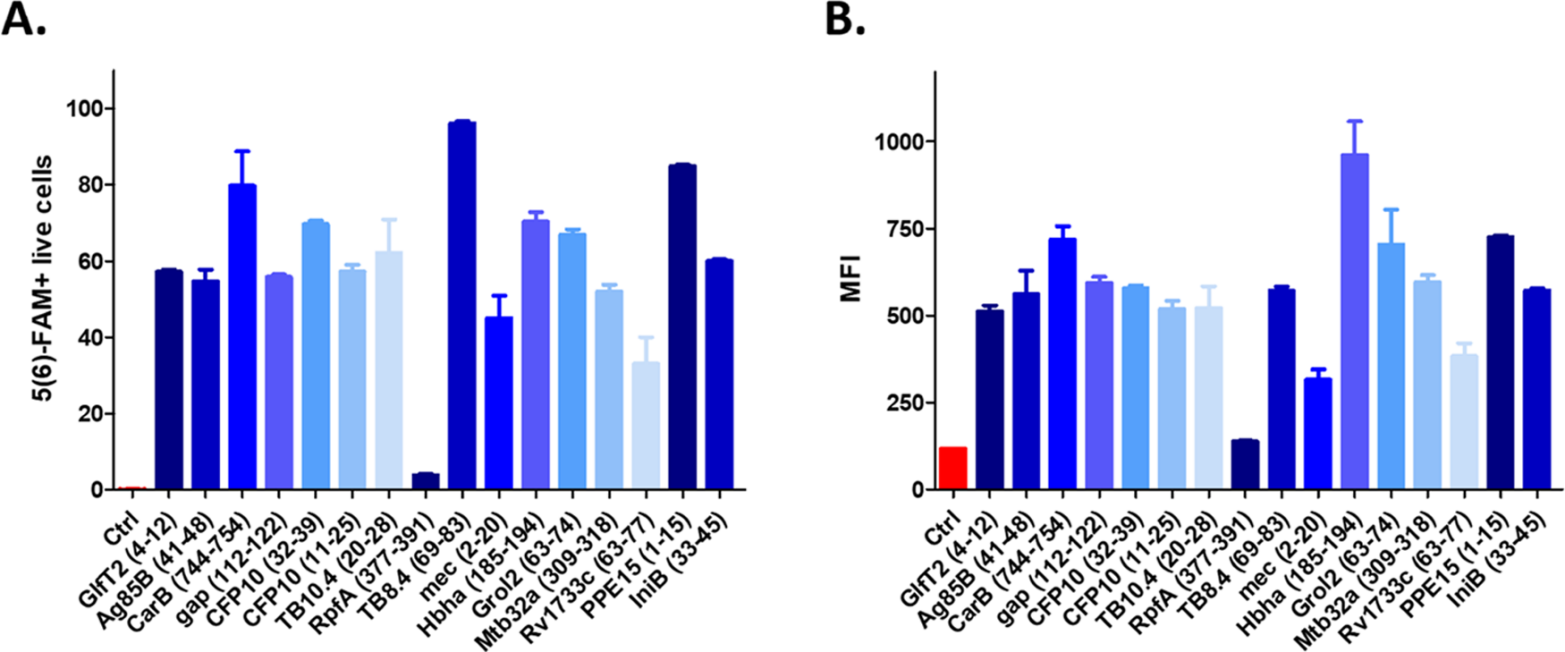
Cellular uptake of 5(6)-FAM-labeled peptides (10 μM) in a MM6 cell line after 2 h of incubation. The percentage of 5(6)-FAM-positive cells (A) and mean fluorescence intensity (MFI) (B) were detected in the FITC channel by a BD LSR II flow cytometer. Each bar is representative of two parallel measurements ± standard error of the mean (SEM). All of the peptides showed a significantly higher internalization rate compare to the medium-treated control cells (*p* < 0.001), except Rv1733c (63–77) (*p* < 0.01) and RpfA (377–391) (*p* > 0.05).

Then, the potential toxicity of the peptides was investigated in MonoMac-6 (MM6), a human monocytic cell line. MM6 is a reliable cell model of mature blood monocytes since it exhibits features like the CD14 marker, phagocytosis, and cytokine production.^37^ Results of the viability assay show that all compounds exhibit an IC_50_ value higher than 100 μM after 24 h of incubation (Figure S5). Also, MM6 cells were used to model the internalization efficiency of the peptides by antigen-presenting cells. Most of the peptides exhibited a UC_50_ value (concentration at which the rate of 5(6)-FAM-positive cells reaches 50%) of approximately 10 μM. On the other hand, a few peptides, such as RpfA (377–391) and Rv1733c (63–77) resulted in a weak internalization profile (Figure 1).

PPE15 (1–15) and IniB (33–45) were selected as promising CD4+ T cell epitopes to include as our vaccine candidate. Despite Grol2 (63–74) showing the highest cell internalization among MHC II binding epitopes, the high homology of the mycobacterial and human heat shock proteins was considered an exclusion factor.^11,38^ CFP10 and Ag85B have been extensively studied as potential targets and are included in the AEC/BC02 vaccine, which is currently in Phase 2a.^39^ CFP10 (32–39) was described as a minimal antigenic epitope recognized by CD8+ T cells from infected mice,^15^ while Ag85B (41–48) was identified as MHC I presented peptide in BCG-infected macrophages.^11^ Likewise, Mtb32a is found in the M72/AS01E vaccine, a late-stage candidate in the TB vaccine pipeline. Mtb32a (309–318) was identified as an MHC I peptide, recognized by cytolytic and IFNγ-secreting CD8+ T cells.^33^ The 10-mer HBHA (185–194) peptide was identified as an MHC II binder in infected macrophages.^11^ However, it could be inferred that the peptide might have a promiscuous binding activity, given its length is suitable for binding the MHC I molecule as well. Moreover, it showed a significant cellular uptake, potentially because of the high lysine content, which could represent an internalization-promoting factor for the final conjugate. GlfT2 (4–12) showed a high IFNγ response in peripheral blood mononuclear cells (PBMCs) from BCG-vaccinated individuals,^11^ and it was selected in this study as a potential MHC I binding peptide.

### Antigen-Recall Assay on Human PBMCs from *Mtb*-Sensitized Donors

A preliminary screening of peripheral blood mononuclear cells (PBMCs) with mycobacteria-specific stimulants was performed to demonstrate prior sensitization of donors. *S*ignificant CD4+ T cell activation was observed in response to stimulation with a purified protein derivative (PPD) and BCG whole-cell lysate (Figure S6). The most promising peptides selected in terms of synthetic feasibility and cellular internalization efficiency were pooled and tested for antigen-specific T cell responses. The composition of each peptide pool was specifically designed to contain MHC I and MHC II binding epitopes that are expressed during either active or latent tuberculosis infection.

Peptide mixture 1 (**PM1**) contained CFP10 (32–39), GlfT2 (4–12), HBHA (185–194), and PPE15 (1–15) peptides, while **PM2** included CFP10 (32–39), Mtb32a (309–318), Ag85B (41–48), and IniB (33–45) peptides. Each peptide was used at a 2 μg/mL concentration in the mixture.

**Figure 2:**
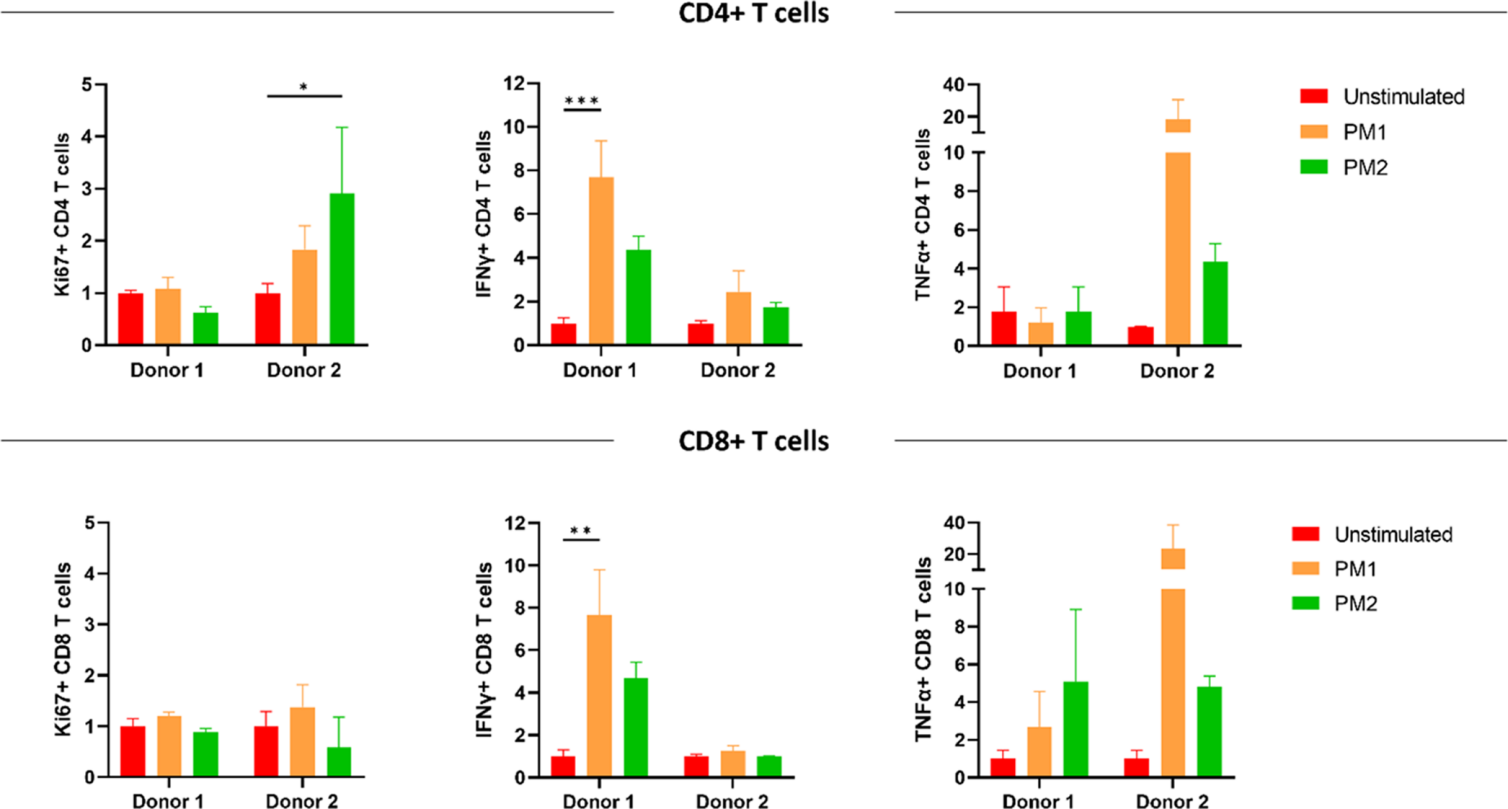
Antigenicity of selected peptide mixtures (**PM1** and **PM2**) with human CD4+ and CD8+ T cells obtained from Mtb-sensitized donors. PBMCs were stimulated for 5 days with the peptide mixtures and then analyzed by flow cytometry for Th1 cytokines (IFNγ and TNFα) and cellular proliferation (Ki67). Data are presented as fold change of the unstimulated control. Statistical analysis was performed using two-way ANOVA, followed by Tukey’s post hoc test; statistical significance: *p < 0.05, **p < 0.01, and ***p < 0.001.

**PM1** stimulation resulted in higher production of IFNγ and TNFα, important cytokines for the control of Mtb infection, although it was not capable of inducing consistent T cell proliferation, in this limited donor (*n* = 2) assay. Overall, **PM1** proved to be more efficient than **PM2** at inducing a Th1 T cell response and CD8+ T cell activation (Figure 2).

### Multiepitope Conjugates: Design and Synthesis

**Figure 3:**
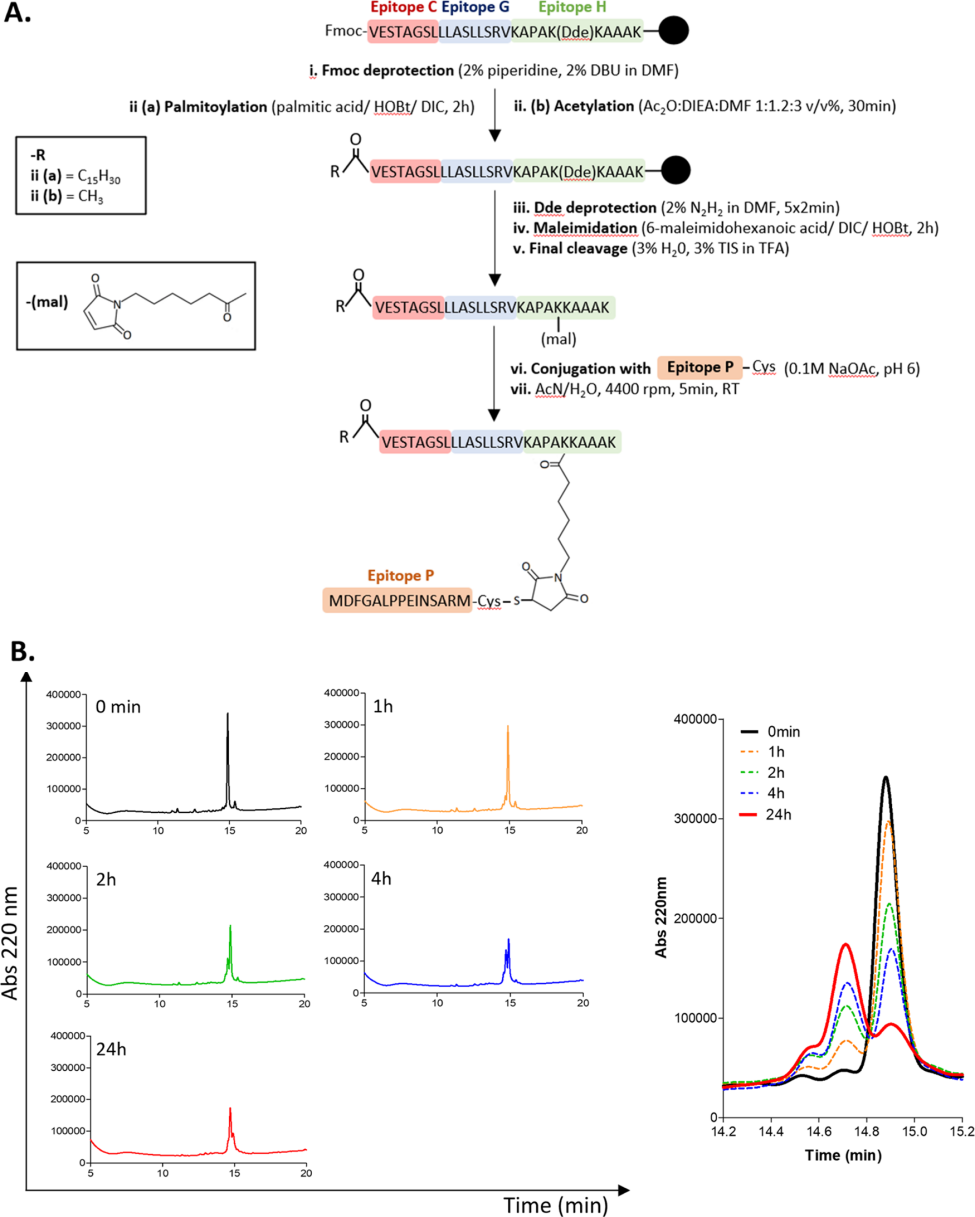
(A) Synthesis outline of multiepitope branched conjugates. CFP10 (32–39) (**Epitope C**), GlfT2 (4–12) (**Epitope G**), and HBHA (185–194) (**Epitope H**) were combined in one linear peptide, synthesized on solid phase. Prior to maleimide derivatization, the linear peptide was either elongated with a 16-carbon fatty acid (ii(a)) or acetylated (ii(b)). The maleimide group was introduced as a 6-maleimidohexanoic acid and coupled through the formation of an amide bond. The derivatives were then reacted with cysteine-elongated PPE15 (1–15) (**Epitope P**) in solution to obtain the final conjugates **Pal-CGHP** and **Ac-CGHP**. (B) Reversed-phase HPLC (RP-HPLC) chromatograms of the conjugation reaction between the linear acetylated peptide and the cysteine-elongated peptide PPE15 (1–15) (left). The reaction mixture was sampled at different time points (0–24 h), diluted with eluents A and B and the retention time was obtained on a Phenomenex Jupiter C12 column with the applied linear gradient 5–100% B, 20 min. The chromatograms were overlapped and maximized to highlight the product formation (right).

Based on the antigenicity profile of the peptide mixtures, **PM1** was selected for the conjugation. As shown in Figure 3A, HBHA (185–194), GlfT2 (4–12), and CFP10 (32–39) were synthesized as a linear chimeric peptide through Fmoc-based SPPS using standard coupling reagents. The N*-*terminus of the linear peptide was either acetylated or palmitoylated (16-carbon fatty acid elongation). A Dde-protected lysine (Lys189) was used to selectively introduce the maleimide group by orthogonal reaction between the ε-amino group of the amino acid and the carboxylic group of 6-maleimidohexanoic acid.

PPE15 (1–15) was elongated by a cysteine residue at the C*-*terminus (**PPE15-Cys**) and then reacted with the maleimide derivative in NH_4_OAc buffer (pH 6) overnight as previously described.^40^ The conjugation was followed over time by analytical RP-HPLC (Figure 3B). Analytical characterization of the obtained conjugates (**Ac-CGHP** and **Pal-CGHP**) is shown in Table S2 and Figures S7, S8.

Chemoselective ligation techniques represent effortless alternatives to produce peptide chimeras compared to other available strategies. For example, synthetic long peptide (SLP) technology could be hampered by synthetic challenges like defective couplings and poor solvation.

The thiol-maleimide reaction is a popular method in bioconjugation since it offers many advantages, such as cost-effectiveness, stability of the thioether bond, and a relatively high reaction rate.

On the other hand, cysteine dimerization can compete with the thiol-maleimide reaction, and experimental measures need to be taken to address this limitation. In this work, the cysteine residue was strategically introduced at the C*-*terminus of the PPE15 peptide because previous results of our research group described it as the most effective arrangement to minimize the disulfide bond formation.^40,41^

The reaction course was estimated by the signal intensity given by the linear maleimide-derivative reagent. The intensity reduction of the peak was approximately 15–25% per each time point (87% at 1 h, 63% at 2 h, 49% at 4 h, and 27% at 24 h). While a buffer at higher pH might have improved the reaction rate, it could have also heightened the risk of side reactions (e.g., dimerization, higher reactivity of free primary amines with the C=C bond of the maleimide, and the ring-opening reaction before thiolation).

Finally, palmitic acid has been associated with enhanced adjuvanticity and in vivo stability of peptide-based therapeutics.^42^ In addition, it has been found to facilitate vaccine uptake in APC due to its ability to anchor the peptide to the cell membrane and facilitate its access to endogenous processing pathways.^43,44^

### Conformation Studies

**Figure 4:**
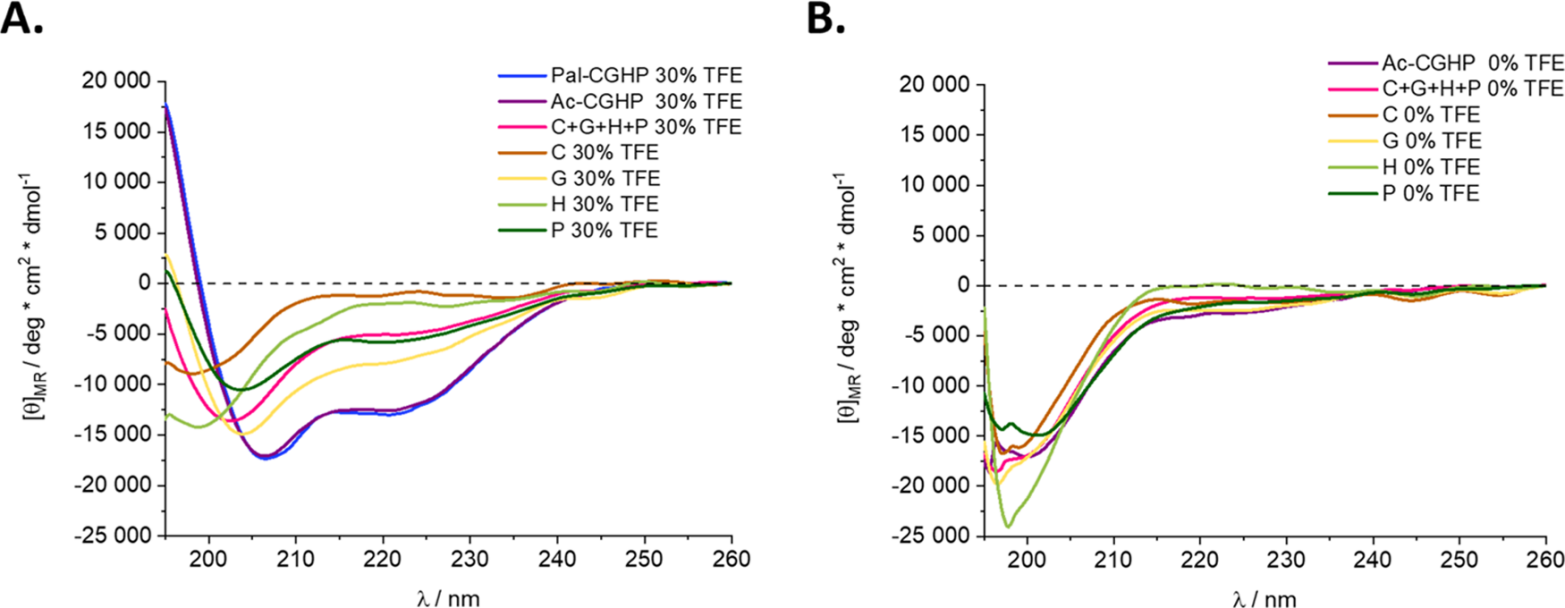
Electronic circular dichroism (ECD) spectra of the compounds in 30% (v/v) TFE in PBS buffer (A). Spectra of single peptides (abbreviated as **C**, **G**, **H**, **P**) and **Ac-CGHP** in PBS (B). The measurements were conducted at pH = 7.4 and the peptide concentration was 26 μM.

The secondary structure of peptides and conjugates was investigated by electronic circular dichroism (ECD) spectroscopy, a recognized method to study conformational changes of the peptide backbone in solution. The conformational arrangements were probed in a physiologically relevant environment (100% phosphate-buffered saline (PBS)) and in a less hydrophilic medium (up to 30% trifluoroethanol (TFE) (v/v) in PBS). With the help of TFE, it is possible to investigate the propensity of a peptide to fold in a hydrophobic environment. The results showed that branched-chain conjugates tended to adopt an ordered structure (e.g., turn or helical) in a non-hydrophilic medium. Also, protecting the N*-*terminus of the conjugate with a palmitoyl group instead of an acetyl group did not alter the secondary structure at the same 30% TFE concentration (Figure 4A). By comparing the secondary structure of short, linear epitope peptides and the branched Ac-CGHP conjugate in a physiological environment, we can conclude that the conjugation did not affect the secondary structure significantly, maintaining the highly dynamic behavior (Figure 4B). Moreover, the conformation of individual peptides and their 1:1:1:1 mixture was examined at varying TFE concentrations. It resulted that peptides were mostly disordered with some turn/helix tendencies at a higher TFE content ratio (Figures S9 and S10). Further experimental details can be found in the Supporting Data.

### Lysosomal Degradation Study of **Ac-CGHP**

**Figure 5:**
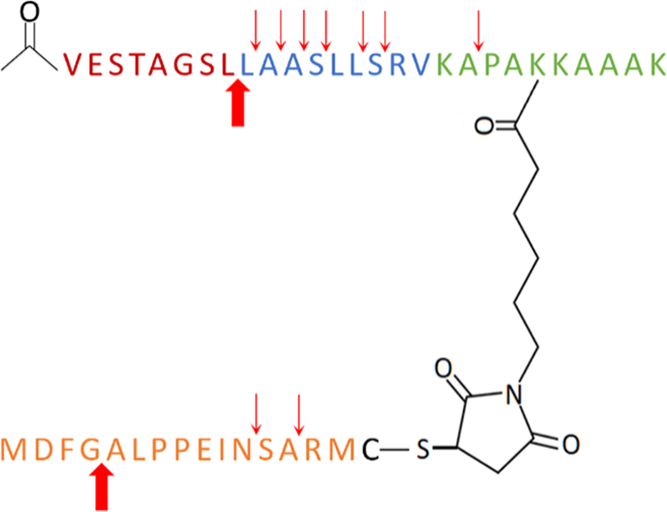
Degradation pattern of **Ac-CGHP** with rat liver lysosomal homogenate. Red arrows indicate the detected potential cleavage sites. The arrows in bold represent the most stable metabolites observed after 4 h incubation.

Antigen presentation highly relies on the endolysosomal compartment, and a proteolytic degradation model can assist in elucidating the generation of MHC I and MHC II ligands.^45^ A lysosomal degradation study, using a rat liver lysosomal homogenate, was performed to mimic the degradation pattern of **Ac-CGHP** and to predict the relationship between antigen proteolysis and overall immunity. **Ac-CGHP** was incubated with the lysosomal homogenate at 37° at acidic pH to facilitate the enzymatic activity, and the conjugate breakdown was investigated at different time points by HPLC-MS (Figures 5, S11 and Table S3). The poor solubility of **Pal-CGHP** rendered it incompatible with these experimental conditions.

Our results pointed out a complete and stable release of CFP10 (32–39) and the formation of the peptide fragment ALPPEINSA as a part of the PPE15 epitope. Although PPE15 (1–15) was previously identified as an MHC II epitope, our bioinformatic analysis (not shown) predicted the ALPEEINSA peptide as a potential MHC I binder, suggesting a promiscuous profile of PPE15 (1–15).

The susceptibility to lysosomal degradation affects the overall immunogenicity of peptide-based vaccines: it was shown that blocking the lysosomal function of APCs antigen presentation can selectively be decreased.^46,47^ Conversely, rapid lysosomal degradation limits the MHC presentation, resulting in a stronger immune response for antigens more resistant to lysosomal proteolysis.^48^

### Internalization Studies Using Confocal Microscopy

Fluorescence confocal laser scanning microscopy and flow cytometry were used to investigate the time-dependent internalization and intracellular localization of **Pal-CGHP**.

RAW264.7 macrophage-like cell line shows stable phenotypic and functional characteristics;^49^ thus, it is frequently employed as an *in vitro* model to study the cellular response to microbes, antigens, and natural products.^50−52^ Due to their good adherence and suitability for microscopic imaging, they were selected as model phagocytic cells.

The lowest 1 μM concentration was chosen to follow **Pal-CGHP** cell internalization since preliminary data evidenced that higher concentrations (5 and 10 μM) were not suitable for imaging due to the oversaturation of the signal (data not shown).

**Figure 6:**
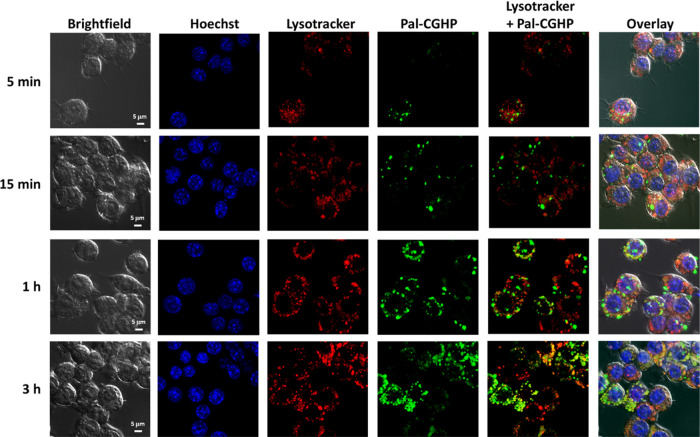
Time-dependent internalization of **Pal-CGHP** (green) into RAW264.7 murine macrophages. Raw264.7 cells were treated with 1 μM **Pal-CGHP** for 5, 15 min, 1, and 3 h. After washing the samples, the lysosomes (red) were visualized with Lysotracker Deep Red and the nuclei (blue) with Hoechst33342 marker for 15 min at 37 °C. Images were taken with a Zeiss LSM510 Meta confocal laser scanning microscope using a 100× oil immersion objective. For the image analysis, NIS-Elements software was used.

The RAW264.7 macrophages were incubated with the **Pal-CGHP** conjugate for 5, 15 min, 1, and 3 h at 37 °C. After removal of the conjugates at these different time points, the cells were labeled with Lysotracker Deep Red, a lysosomal marker, together with Hoechst33342 nuclear marker for 15 min at 37 °C. The macrophages were then rinsed with OptiMem medium and immediately processed in a fluorescence imaging experiment (Figure 6).

Flow cytometry data showed a time-dependence **Pal-CGHP** uptake, reaching its maximum after 1 h incubation (Figure 6).

**Figure 7:**
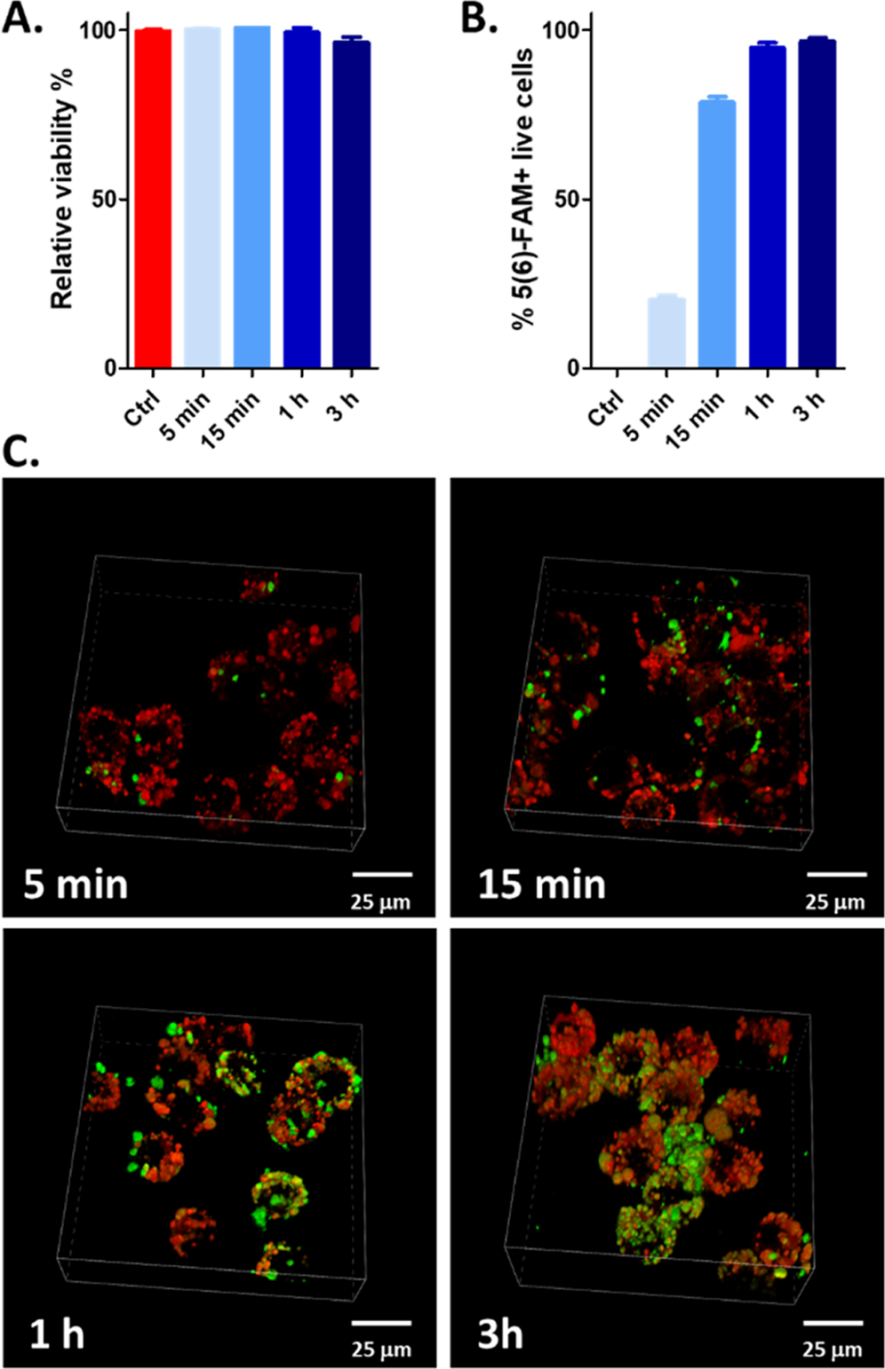
**Pal-ACGHP** association with Lysotracker Deep Red endolysosomal stain. Maximum intensity projection 3D plots showing colocalization (yellow inclusions) of **Pal-CGHP** (green) with the Lysotracker Deep Red (red) in RAW264.7 murine macrophages from 1 h time point (C). 3D images were prepared from maximum intensity of projection Z-scans using Nikon NIS-Elements software. In addition to microscopic imaging, flow cytometry was also employed to measure the relative viability and cellular uptake in RAW264.7. The **Pal-CGHP** conjugate was not toxic to the cells (A) and showed a time-dependent internalization rate (B). After 5, 15 min, 1, and 3 h, the percentage of 5(6)-FAM-positive live cells was significantly higher (*p* < 0.001) compared to the negative control. Statistical analysis was performed using one-way ANOVA, followed by Tukey’s post hoc test.

After as little as 5 min of incubation, the **Pal-CGHP** conjugate was detected in the RAW264.7 macrophages, suggesting endosomal compartmentalization (Figure 6). Furthermore, an increase in the fluorescence signal derived from **Pal-CGHP** was detected after 15 min treatment, but no colocalization with the Lysotracker Deep Red stain was observed (Figure 6). However, 1 h and more prominently 3 h long incubation resulted in an increase in the colocalization of the conjugate and the endolysosomal marker, as depicted in the three-dimensional (3D) images shown in Figure 7C. The time dependence of the cellular uptake seen in the 3D microscopic images was supported by the results of the flow cytometric measurement (Figure 7A,B), consistently with the literature data showing the lysosomal association with lipopeptides after 1 h treatment in antigen-presenting cells.^50^

### Investigation of the Cellular Uptake of **Ac-CGHP** and **Pal-CGHP** on Different Cell Types

Parallel with RAW264.7, two other immunocompetent cell models were investigated, the murine bone-marrow-derived dendritic cells (BMDC) and MM6 cells. Bone-marrow-derived CD11c+ cells have strong phagocytic ability and play a pivotal role in antigen processing and presentation.^51^

**Figure 8:**
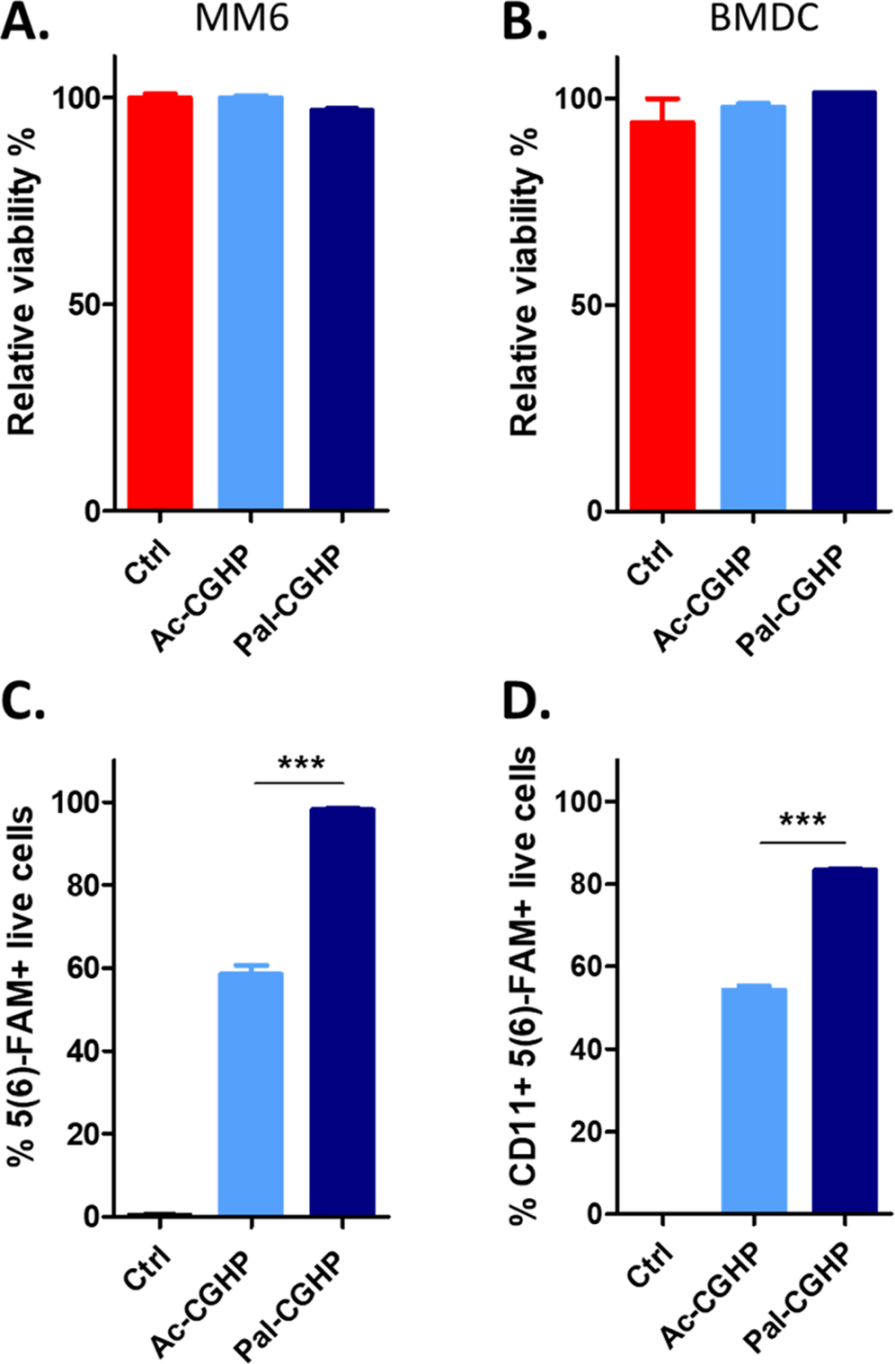
Cytotoxicity (A, B) and cellular uptake (C, D) of **Ac-CGHP** and **Pal-CGHP** were investigated in MM6 cell line and murine BMDC using flow cytometry after 2 h of incubation. Cell viability was expressed as a comparison to untreated cells (relative viability %). Each bar represents a mean value of a triplicate ± SEM. Statistical analysis was performed by one-way ANOVA, followed by Tukey’s post hoc multiple comparison. Statistical significance: *** *p* < 0.001.

The uptake rates of MM6 and BMDC cells were consistent with the data of RAW264.7 macrophages. After 2 h of incubation, 98 and 83% of the cells were positive for the **Pal-CGHP** conjugate, respectively (Figure 8C,D). A significantly lower internalization rate was measured for the acetylated conjugate **Ac-CGHP**, demonstrating that the palmitoylation greatly facilitates cell entry. At the measured 10 μM concentration, none of the conjugates were cytotoxic to the cells (Figure 8A,B).

### Transwell Cell Culture Experiment

First, we studied the penetration ability of **Ac-CGHP** and **Pal-CGHP** conjugates into epithelial cells (Calu-1, Vero-E6, and H838). Efficient cellular uptake was measured in all cells, especially for the palmitoylated conjugate. More than 50% of the cells were 5(6)-FAM-positive after 45 min of incubation with as low as 5 μM **Pal-CGHP** concentration. To investigate how peptides interact with tissue barrier models, we developed a submerged, noncontact monolayer setup, representing different epithelial barriers.^52,53^

Transwell inserts are permeable supports that fit well into common 24-well cell culture plates. They feature a porous membrane, and the inserts provide a simple support for culturing monolayers to mimic tissue barriers. The membrane permits the cellular uptake of the peptides and facilitates metabolic activities that mimic conditions occurring in *in vivo* environments.

**Figure 9:**
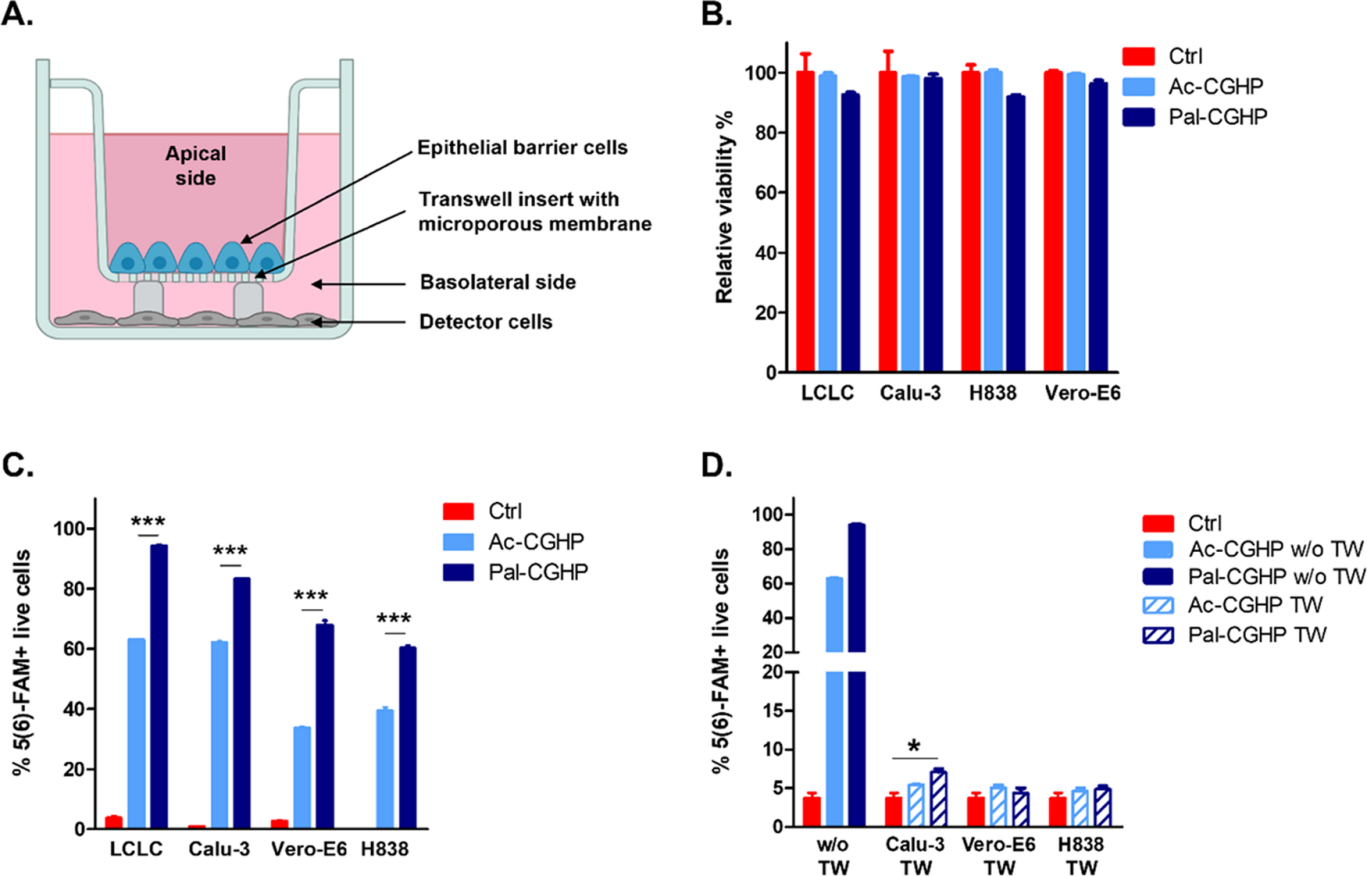
Schematic representation of the submerged transwell noncontact coculture arrangement containing epithelial barrier cells on polycarbonate microporous insert and detector cells on the basolateral chamber’s bottom (A). Cytotoxicity (B) and penetration ability (C) of **Ac-CGHP** and **Pal-CGHP** conjugates into different epithelial cells. The viability of the treated cells was compared to the untreated controls (relative viability %, B). Percentage of peptide-positive epithelial cells is shown (C) after 45 min treatment with 5(6)-FAM-labeled conjugates at 5 μM concentration. Flow cytometric analysis of the LCLC-103H detector cells with and without transwell inserts are represented as the percentage of 5(6)-FAM-positive cells, after adding the peptides to the apical side at 5 μM concentration (D).

After treatment with 5(6)-FAM-labeled conjugates, we observed no monolayer damage, and therefore, no fluorescence signal occurred by the detector cells at the basolateral side (Figure 9). This indicates that exposure to the active peptide did not cause any changes in the monolayer integrity. Based on the uptake results, we can conclude that the conjugates do not translocate freely across the epithelial barrier but appear to internalize and retained within the cells.

### Immunological Evaluation

**Figure 10:**
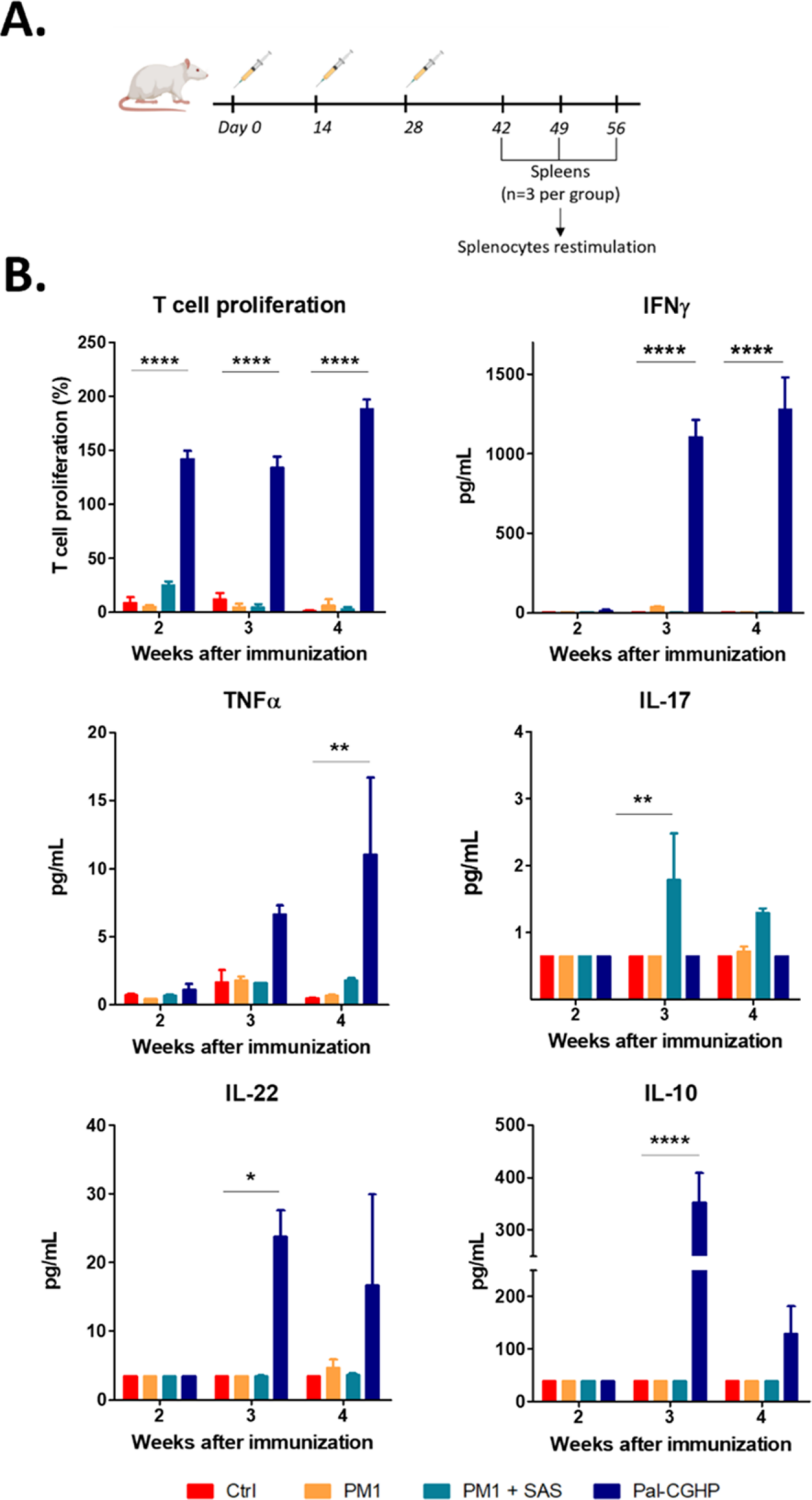
Immunization timeline (A) and evaluation of the immune response in mice isolated splenocytes (B). T cell proliferation was assessed by AlamarBlue assay, and the cytokine production was measured by LEGENDplex bead-based immunoassay. Represented data are mean ± SEM. (*n* = 3 mice/group). Statistical analysis was performed using one-way ANOVA followed by Tukey’s post hoc test; statistical significance: *p < 0.05, **p < 0.01, and ****p < 0.0001.

The **Pal-CGHP** conjugate, the peptide mixture **PM1** in PBS, and **PM1** formulated with the Sigma Adjuvant System (SAS) were administered subcutaneously to BALB/c mice three times, 2 weeks apart (Figure 10A). We then measured antigen-specific splenic T cell proliferation and cytokine secretion among immunized and unimmunized animals (Figure 10B).

As expected, **PM1** did not produce a noticeable immune response, likely due to intrinsic poor immunogenicity and *in vivo* stability of short synthetic peptides. Interestingly, the SAS formulation did not improve the overall immunogenicity of the peptide mixture. Despite the two main components of the adjuvant, monophosphoryl lipid A and the trehalose dicorynomycolate, being widely known inducers of type-1 CD4+ T helper cell immune responses,^54,55^ failed to induce appreciable IFNγ production. SAS formulation improved IL-17 production, a crucial proinflammatory cytokine in the control of *Mtb* infection^56^ but not IL-22. This could be due to the IL-17 concentration being too low to trigger significant IL-22 levels. **Pal-CGHP** immunization produced significant T cell proliferation, which slightly increased over time, with the highest levels observed 4 weeks post-immunization. Similarly, IFNγ and TNFα concentrations followed a time-dependent increase. In contrast to the SAS-formulated candidate, **Pal-CGHP** induced significant IL-22 production but no IL-17 response. Recently, IL-22 was suggested to be associated with protection during TB infection,^57−59^ while IL-10 was proposed to modulate the Th1 proinflammatory immune response and prevent potentially harmful effects of an excessive immune response. The high levels of IL-10 observed in this study may be related to the strong IFNγ response.^60^

When splenocytes from **Pal-CGHP** immunized mice were restimulated with the individual C/G/H/P peptides, bearing the original sequences, epitope-specific antigenicity was observed (Figure S12), suggesting that their chemical modification occurring in the conjugation did not compromise the epitope recognition.

Thus, the results suggest that **Pal-CGHP** is more promising than the two tested candidates and it merits further investigation as a multiepitope self-adjuvanting vaccine platform.

## Conclusions

Although no peptide-based vaccine against *Mtb* has progressed to the clinical stage, preclinical research in the field is expanding in different areas, including epitope screening and prediction, vaccine design, and the assessment of immunogenicity and efficacy in animal models.^9^

Choosing T cell epitopes that elicit a pathogen-specific immune response is crucial to developing effective and safe peptide vaccines. The advent of bioinformatics technology has significantly contributed to this, albeit the validation of the predicted epitopes remains essential, especially in the case of a complex pathogen like *Mtb*.

This work presents an accurate selection process starting from previously validated T cell epitopes and finalized using a recognized method like the *ex vivo* antigen-recall assay on *Mtb*-sensitized PBMCs.^61^ Our results pointed out that the peptide pool **PM1** successfully stimulated the production of IFNγ and TNFα, pivotal cytokines in the control of TB, and supported the published evidence on the immunogenicity of the contained single peptides.^11,15^

Since the actual activity of synthetic peptides *in vivo* is affected by their intrinsic weaknesses, such as low *in vivo* stability and poor immunogenicity, a proper formulation is needed. Different approaches are suggested in the literature to target these drawbacks, which involve mainly adjuvanted delivery systems or the formation of nanostructures through a self-assembly process.^62,63^

In our study, the formulation with the SAS emulsion did not improve the immunogenicity of **PM1**, conversely to previous evidence where it proved to enhance the effectiveness of subunit vaccines in terms of both T cell and antibody response.^55,64−66^

An alternative method, including the one presented in this work, is assembling more complex structures.

For example, SLP technology combined with immunostimulatory molecules has been suggested to extend the antigen presentation process in time and improve immunogenicity.^67−69^ The synthetic limitations that might occur with this approach above a given length cut-off could be addressed using chemical conjugation techniques.^62^ In this regard, thiol-maleimide chemistry proved to be an efficient, cost-effective, and user-friendly approach to developing multiepitope branched conjugates.^40,70^

The discovery that lipid incorporation improved the immunogenicity of synthetic peptide-based vaccines has led to the development of self-adjuvanting lipopeptides.^71^ Beyond this, they show other advantages, described by Wiesmüller et al., and include compatibility with SPPS, higher stability, and enhanced cellular uptake.^72^ This latter feature was observed with the palmitoylated conjugate **Pal-CGHP**, which proved a significantly higher internalization over **Ac-CGHP**.

The exact mechanism by which palmitoylation affects the process remains unclear. While di- or tripalmitoylation (e.g., Pam_n_Cys lipopeptides) is associated with TLR-associated internalization,^42^ other studies pointed out that single-chain saturated fatty acids may not follow this pathway but efficiently integrate into the APCs’ membranes and undergo receptor-independent internalization.^73,74^

Also, the results shown by **Pal-CGHP** in our immunization study are consistent with previously described examples of palmitoylated peptide-based vaccine candidates against different pathogens, including HIV, malaria, and *Mtb*.^75−77^ To date, there is limited knowledge about how palmitic acid impacts the immunological profile of lipopeptide vaccine candidates.^44^

In conclusion, we described the design and development of an immunogenic self-adjuvanting multistage vaccine platform obtained by combining the synthesis of a multiepitope lipopeptide along with chemoselective ligation.

From a future perspective, conducting a study using a TB challenge model will be crucial to assess the effectiveness of our vaccine platform. In this regard, alternative *in vitro* assays have been introduced to overcome the ethical, cost, and logistical obstacles of *in vivo* testing, such as the direct mycobacterial growth inhibition assay (MGIA).^78^ Thus, it can offer an undemanding and more cost-effective way to determine the early protective efficacy of our candidate, with the additional advantage of maximizing the 3Rs principle (replacement, reduction, and refinement of animals used in research).

## Experimental Procedures

### Reagents

Amino acid derivatives and piperidine were obtained from Iris Biotech (Marktredwitz, Germany). *N,N*′-diisopropylcarbodiimide (DIC), 1-hydroxybenzotriazole (HOBt), 1,8-diazabicyclo[5.4.0]undec-7-ene (DBU), hydrazine monohydrate (N_2_H_2_·H_2_O), triisopropylsilane (TIS), ammonium acetate (NH_4_OAc), palmitic acid, 6-maleimidohexanoic acid, acetic anhydride (Ac_2_O), formic acid, Fmoc-Rink Amide MBHA resin, and 5(6)-carboxyfluorescein (5(6)-FAM) were obtained from Merck (Budapest, Hungary). Trifluoroacetic acid (TFA), *N,N*-dimethylformamide (DMF), dichloromethane (DCM), diethyl ether, and acetonitrile (AcN) were from VWR (Budapest, Hungary).

For the biological assays, RPMI-1640, Dulbecco’s modified Eagle’s medium (DMEM), PBS, 2 mM l-glutamine, and trypan blue were from Lonza (Basel, Switzerland), while trypsin, nonessential amino acids, and penicillin–streptomycin were from Gibco (Thermo Fisher Scientific, Waltham, MA). Fetal bovine serum (FBS) was from EuroClone (Pero, Itlay); HPMI buffer (9 mM glucose, 10 mM NaHCO_3_, 119 mM NaCl, 9 mM HEPES, 5 mM KCl, 0.85 mM MgCl_2_, 0.053 mM CaCl_2_, 5 mM Na_2_HPO_4_ × 2H_2_O, pH = 7.4) was prepared in-house using components obtained from Merck (Budapest, Hungary). For the AlamarBlue assay, resazurin sodium salt (Merck) was dissolved in PBS (0.15 mg/mL, pH 7.4) and sterile-filtered. Sigma Adjuvant System (SAS) was from Merck (Budapest, Hungary).

### Peptide Synthesis

Peptides were synthesized on solid phase (Fmoc-Rink Amide MBHA resin, capacity = 0.51 mmol/g) by Fmoc/*^t^*Bu strategy with DIC/HOBt coupling reagents manually or in a Syro-I automated peptide synthesizer (Biotage, Uppsala, Sweden). Fluorescently labeled derivatives were obtained by coupling 5(6)-carboxyfluorescein (5(6)-FAM) at the N-terminus using the DIC/HOBt coupling method. Palmitoylated derivatives were produced by coupling palmitic acid to the N-terminus of the peptide using the DIC/HOBt coupling method. When it was needed, the peptide was acetylated by reacting the N*-*terminal amino group with Ac_2_O/DIEA/DMF = 1:1.2:3 (v/v) for 2 + 30 min. Peptides were cleaved with TFA using H_2_O:TIS (3–3%, v/v) as scavengers (3 h, RT). After filtration, crude products were precipitated in cold diethyl ether, centrifuged (4000 rpm, 5 min), and freeze-dried. Peptides were purified by RP-HPLC on a semipreparative Phenomenex Jupiter Proteo C12 column (10 μm, 90 Å, 10 mm × 250 mm) with linear gradient elution using 0.1% TFA in water (eluent A) and 0.1% TFA in AcN/water = 80:20 (v/v) (eluent B) performed on an UltiMate 3000 Semiprep HPLC (Thermo Fisher Scientific, Waltham, MA). For the palmitoylated derivative, a C4 Phenomenex Jupiter (250 mm × 10 mm) column was used. Purified peptides were analyzed by RP-HPLC (LC-40 HPLC System from Shimadzu, Kyoto, Japan) on an analytical C12 column using gradient elution with the eluents A and B (flow rate was 1 mL/min, the gradient was 5–100 B% in 20 min, UV detection at λ = 220 nm). The molecular mass of the peptides was determined using a Thermo Scientific (Waltham, MA) Q Exactive Focus Hybrid Quadrupole-Orbitrap Mass Spectrometer. Parameters: capillary voltage: 3.5 kV, flow rate: 0.300 mL/min, nebulizer gas: 11.25 psi, heated capillary temperature: 256 °C.

Branched conjugates (**Ac-CGHP** and **Pal-CGHP**) were produced following a previously described synthetic route.^40^ Briefly, DDe orthogonal protecting group of lysine, inbuilt in the sequence of **Ac-CGH** or **Pal-CGH**, was removed selectively by treating the resin with 2% N_2_H_2_ in DMF (6 × 1 min). Then, the free amino group was allowed to react with 6-maleimidohexanoic acid in the presence of DIC/HOBt coupling reagents (2 h, RT). After the completion of the reaction, **Ac-CGH(mal)** and **Pal-CGH(mal)** derivatives were cleaved from the resin and purified by HPLC as described above. Maleimide-derived **Ac-CGH(mal)** or **Pal-CGH(mal)** (20 mg) were dissolved in 7 mL of a 1:6 mixture of DMF and 0.1 M NH_4_OAc (pH 6). Subsequently, 1.2 equiv of cysteine-elongated PPE15 epitope (**PPE15-Cys**) were slowly added to the solution. The mixture was stirred overnight, then freeze-dried, purified by HPLC, and characterized as mentioned above.

### Electronic Circular Dichroism (ECD) Spectroscopy

ECD measurements were performed on a Jasco J-1500 dichrograph from Jasco Corporation, Japan. The measurements were performed in a 0.1 cm quartz cell over the wavelength range of 195–260 nm at a temperature of 25 °C. Each ECD spectrum was obtained as an average of five individual scans. The solvent reference spectrum was used as baseline, which was automatically subtracted. Band intensities were expressed as mean residue ellipticity ([Θ]_MR_/(deg × cm^2^ × dmol^–1^)) by correcting the recorded spectra according to the individual peptide sample concentration, which was determined from the extinction coefficient and absorbance at 214 nm (https://www.protpi.ch/Calculator/ProteinTool). Sample preparation was obtained as follow: **Pal-CGHP** was tested at a concentration of 0.125 mg/mL (∼26 μM) in various mixtures of TFE and PBS, increasing the TFE concentration from 15 to 40% in increments of 5%. **Ac-CGHP** conjugate and individual peptides (CFP10, GlfT2, HBHA, PPE15) were also assessed at the same concentration (∼26 μM) by varying the TFE concentration from 0 to 30% in increments of 5%. All of the conjugates and peptides were diluted from a stock solution of 104 μM before testing.

### Degradation Study of Ac-CGHP in Rat Liver Lysosomal Homogenate

The rat liver lysosomal homogenate preparation and the protein content were obtained as previously described.^79^ The protein concentration was 71.76 μg/μL. The lysosomal homogenate was diluted in a 0.2 M NaOAc buffer (pH 5) solution to have a final protein concentration of 0.22 μg/μL. **Ac-CGHP** was added to the solution to reach a lysosomal protein/peptide concentration ratio of 1:20 m/m. The mixture was stirred at 600 rpm at 37 °C, and 50 μL aliquots were sampled at different time points (0, 15, 30 min, 1, 4 h). The enzymatic activity was quenched by adding 5 μL of formic acid to the samples. Then, samples were immediately frozen at −25 °C. **Ac-CGHP** (0.011 μg/μL) dissolved in the buffer was used as a negative control. Control samples were taken at 0 min and 4 h time points. All of the samples were analyzed by HPLC-MS on a Q Exactive Focus Hybrid Quadrupole-Orbitrap Mass Spectrometer.

### Cells

MonoMac-6 (MM6) human monocytic cell line^37,80^ (DSMZ no. ACC 124) was maintained as an adherent culture in RPMI-1640 supplemented with 10% FBS and with 2 mM l-glutamine, 1% nonessential amino acids, and 1% penicillin–streptomycin (from 10,000 units of penicillin and 10 mg of streptomycin/mL) at 37 °C in a humidified atmosphere containing 5% CO_2_.

LCLC-103H human lung large cell carcinoma (DSMZ no. ACC 384),^81,82^ Calu-1 human epidermoid carcinoma (Sigma 93120818),^83,84^ H838 human lung adenocarcinoma (ATCC CRL-5844),^85^ and Vero-E6 African green monkey (*Chlorocebus sabaeus*) kidney epithelium cells (ECACC 85020206) were maintained in DMEM medium supplemented with 10% FBS and with 2 mM l-glutamine, 1% nonessential amino acids, 1 mM sodium pyruvate, and 1% penicillin–streptomycin (from 10,000 units of penicillin and 10 mg of streptomycin/mL).

Mouse bone-marrow-derived dendritic cells (BMDC) were obtained from tibiae and femurs of 8–10-week-old BALB/c mice. Isolated cells were kept in a cryopreservation solution consisting of 90% FBS with 10% DMSO at −80 °C. For the experiment, on day 0, bone-marrow cells were cultured in RPMI-1640 media containing 10% FBS, 20 ng/mL GM-CSF, and 10 ng/mL IL-4 in an ultralow attachment flask (Corning). Half of the media was replaced every 3 days, and on day 7, cells were collected and plated in a 24-well ultralow attachment plate (Corning) for further analysis. The purity of the BMDC was determined by flow cytometry using APC anti-mouse CD11c monoclonal antibody (Biolegend, Cat no. 117309). The percentage of CD11c-positive cells was always above 70%.

### Cytotoxicity and Cellular Uptake *In Vitro* Investigation

Cytotoxicity of single peptides was investigated on MM6 human cells using the AlamarBlue viability assay. Cells were seeded in a 96-well tissue culture plate (Sarstedt, 2 × 10^4^ cells/200 μL medium) and the day after, they were treated with the peptides (100 μM) in serum-free media for 24 h. After centrifugation (1000 rpm, 5 min) and washing with serum-free medium, cell viability was estimated by adding 22 μL of AlamarBlue reagent (resazurin sodium salt, 0.15 mg/mL, dissolved in PBS, pH 7.4). After 2 h of incubation, the fluorescence was detected (λ_Ex_ = 530/30 and λ_Em_ = 610/10 nm) in a Synergy H4 multimode microplate reader (BioTek, Winooski, VT). All measurements were performed in quadruplicate and the mean viability, compared to the untreated control was presented together with ± SEM.

The cellular uptake of 5(6)-FAM-labeled peptides was investigated in the MM6 cell line. 5 × 10^4^ cells were seeded in a 24-well tissue culture plate (Sarstedt) in complete media 2 days prior to the experiment. Then, cells were treated with peptides at 10 μM concentration for 2 h. After two washes, cells were detached using 0.25% trypsin solution (100 μL/well, 37 °C, 5 min), then the reaction was stopped by adding 0.8 mL of HPMI medium supplemented with 10% FBS. Cells were washed with HPMI, resuspended in 0.3 mL of HPMI medium, and the intracellular fluorescence intensity was investigated on a BD LSR II flow cytometer (BD Biosciences, San Jose, CA) on a FITC channel (emission at λ = 505 nm). External fluorescence was quenched by adding 10 μL of 0.04% trypan blue solution. Data were analyzed with FACSDiva 5.0 software (BD Biosciences, San Jose, CA). All of the measurements were performed in triplicate and the mean percentage of 5(6)-FAM-positive cells together with SEM were graphically presented.

The cytotoxicity and the internalization rate of the branched conjugates were also investigated on BMDCs by flow cytometry. BMDCs were seeded in a 24-well ultralow attachment plate (250.000 cells/300 μL RPMI-1640 medium, containing 10% FBS), 1 day prior to the experiment. Cells were treated with 5(6)-FAM-labeled conjugates at 10 μM concentration for 2 h. Then, the cells were washed with serum-free medium and transferred to FACS tubes. After centrifugation, Fc receptors were blocked with TruStain FcX-PLUS (0.25 μg/100 μL, Biolegend #156603) for 10 min in the dark (4 °C), then stained with CD11c-APC (Biolegend #117309, 0.25 μg/100 μL, 20 min, 4 °C), or with the isotype control (Biolegend 400911). Cells were washed with staining buffer and then analyzed by flow cytometry.

Cellular uptake and localization of **Pal-CGHP** in murine RAW264.7 macrophages were followed using a confocal laser scanning microscope. Cells were cultured in high glucose containing DMEM medium supplemented with 10% FBS and 1% penicillin/streptomycin (P/S) at 37 °C, 5% CO_2_ and subcultured at regular intervals. Cells were seeded 1 day prior to the experiments on 30 mm coverslips (thickness 1, Assistant, Karl Hecht GmbH, Sondheim vor der Rhön, Germany) at a density of 650,000 cells/2 mL complete DMEM medium in a 35 mm culture dish. On the following day, cells were treated with **Pal-CGHP** at a concentration of 1 μM in complete medium for different time intervals (5, 15 min, 1, and 3 h) at 37 °C, 5% CO_2_. Before adding the peptide to the cells, **Pal-CGHP** was sonicated for 15 min. After removal of extracellular **Pal-CGHP** by rinsing the cell monolayer, macrophages were immediately stained with LysoTracker Deep Red (Invitrogen, Waltham, MA) at 50 nM of final concentration in the presence of Hoechst33342 (1 μg/mL) nuclear stain in complete DMEM for 15 min at 37 °C, 5% CO_2_. After three washing steps, colorless Opti-MEM was used as an imaging medium. Images were taken immediately with an LSM510 Meta confocal laser scanning microscope (Zeiss, Munich, Germany) using a 100× oil immersion objective with the following settings: ex 403 nm/em 450 nm (blue), ex 488 nm/em 525 nm (green), and ex 643 nm/em 700 nm (deep red). NIS-Elements software was used for both image acquisition and analysis.

The cytotoxicity and internalization rate of the **Pal-CGHP** conjugate were also investigated on RAW264.7 murine macrophages by flow cytometry. Raw264.7 cells were seeded on a 24-well plate (100.000 cells/300 μL DMEM medium, containing 10% FBS and 1% P/S), 1 day prior to the experiment. Cells were treated with the 5(6)-FAM-labeled conjugate at 1 μM concentration for 5, 15 min, 1, and 3 h. After two washes, cells were detached using 0.25% trypsin solution (100 μL/well, 37 °C, 5 min), then the reaction was stopped by adding complete DMEM medium. Cells were washed with OptiMem medium, resuspended in 0.1 mL OptiMem supplemented with 2% FBS and the intracellular fluorescence intensity was investigated on a CytoFLEX LX flow cytometer (Beckman Coulter, Vienna, Austria) on an FITC channel (emission at λ = 505 nm). Data were analyzed with Cytexpert software (Beckman Coulter, Vienna, Austria).

### Antigen-Recall Assay on Human PBMCs from *Mycobacteria*-Sensitized Donors

Two blood donors from the hospital blood bank were identified as immunoreactive to PPD in a standard T cell recall assay, though we were not able to discriminate whether this was due to latent MTB infection or BCG immunization, due to restrictions policy governing the use of these tissues. Peripheral blood mononuclear cells were obtained from whole blood through density centrifugation using Histopaque-1077 (Sigma-Aldrich) as per the manufacturer’s recommendations. PBMCs were cryopreserved at −80 °C using FBS with 10% (v/v) DMSO. Revived PBMCs used in all assays had a viability of >90%.

For antigen-recall assays, PBMCs (1 × 10^6^ cells/well) were plated in flat-bottom 96-well plates in RPMI-1640 media supplemented with 10% FBS (v/v), 100 U/mL penicillin, 100 μg/mL streptomycin, 5 mM l-glutamine, 50 μM β-mercaptoethanol, and 10 mM 4-(2-hydroxyethyl)-1-piperazineethanosulfonic acid (HEPES) buffer (Sigma). Cells were then stimulated with the peptide mixtures (final peptide concentration was 2 μg/mL), *Mtb* PPD (5 μg/mL), *Mycobacterium bovis* BCG whole-cell lysate (5 μg/mL), or PHA (5 μg/mL). After 5 days, cells were washed with PBS and surface stained with a master mix of anti-human Fc-receptor blocking antibodies (TruStain FcX, diluted 1:250), fixable viability dye (Zombie Red Fixable Viability, diluted 1:500), Brilliant Violet 421-conjugated CD3, PerCP/Cy5.5-conjugated CD4, and Brilliant Violet 510-conjugated CD8 antibodies (all from BioLegend and diluted 1:200) for 45 min, 4 °C. Fixation and permeabilization of cells was done using eBioscience Foxp3/Transcription Factor Staining Buffer. This was followed by intracellular staining with Alexa Fluor 647-conjugated Ki67, Alexa Fluor 700-conjugated IFN-γ, and PE-Cyanine7 TNFα antibodies (all from BioLegend and diluted 1:200). Cell acquisition was performed using a CytoFLEX S flow cytometer (Beckman Coulter) and analyzed using FlowJo v10 (BD Life Sciences, Ashland, OR).

### Transwell Experiment

As control, the internalization rate of 5(6)-FAM-labeled **Ac-CGHP** and **Pal-CGHP** conjugates was measured on LCLC-103H, Calu-1, Vero-E6, and H838 cells using flow cytometry. Cells were seeded in a 24-well plate 1 day prior to the experiment and treated with the compounds at 5 μM final concentration for 45 min. Cells were then washed twice with incomplete DMEM medium and detached using 0.25% trypsin solution (100 μL/well, 37 °C, 5–10 min). The reaction was stopped by adding 0.8 mL of HPMI medium supplemented with 10% FBS, then cells were washed and resuspended in 0.3 mL of HPMI medium. The intracellular fluorescence intensity was investigated on a BD LSR II flow cytometer.

For barrier assays, epithelial cell (Calu-1, Vero-E6, and H838) suspensions were added into the apical chamber (7.5 × 10^4^ cells in 300 μL complete DMEM medium) and seeded on a polycarbonate transwell insert (Nunc, Sigma), exhibiting a growth area of 0.5 cm^2^ and a pore size of 0.4 μm. At the same time, 500 μL of the medium were added to the basolateral side. On day 4, the medium was changed, and epithelial cells were grown up to confluence, which was checked prior and after the experiments with CellTracker Green (CMFDA (5-chloromethylfluorescein diacetate), Invitrogen, C2925, data not shown, details see in refs (52, 53)). On day 4, detector cells (LCLC-103H) were seeded on a 24-well plate (1 × 10^5^ cells in 1000 μL complete DMEM medium) and on day 5, the transwell inserts were placed above the detector cells (basolateral position). The FAM-labeled **Ac-CGHP** and **Pal-CGHP** conjugates were added to the apical side at 5 μM final concentrations. The cultures were incubated for 45 min, then the transwell inserts were removed and the detector cells were processed as described in the section of cellular uptake studies and analyzed by a BD LSR II flow cytometer.

### Ethical Statement

Animal experiments were carried out in accordance with the guidelines of EU Directive 2010/63/EU and Hungarian laws and were approved by the Hungarian Scientific Ethical Committee on Animal Experimentation under the protocol number PE/EA/2569-4/2016.

Human blood was obtained from healthy volunteers from the National Health Service Blood and Transplant Unit (NHSBT) at St. George’s Hospital London under ethical approval SGREC16.0009.

### Mice Immunization and Splenocytes Preparation

6–8-week-old BALB/c mice were immunized with the vaccine candidates (100 μM peptide concentration in 100 μL of PBS) subcutaneously three times, 2 weeks apart. Vaccine formulations using the Sigma Adjuvant System (containing 0.5 mg of monophosphoryl lipid A (detoxified endotoxin) from *Salmonella minnesota* and 0.5 mg of synthetic trehalose dicorynomycolate in 2% oil (squalene)–Tween 80–water) were made following the manufacturer’s instructions. Three groups of mice (*n* = 3) were euthanized 2, 3, and 4 weeks after the last immunization. Spleens were removed under sterile conditions and single splenocyte suspensions were prepared by mashing the cells through a 70 μM cell strainer (Falcon, Corning, NY). After the lysis of the red blood cells with RBC lysis buffer (5 mL, diluted from Red Blood Cell Lysis Buffer 10×, Biolegend), the cells were washed and resuspended in 10% FBS containing RPMI-1640 media.

### T Cell Proliferation and Cytokine Production Assays

For the investigation of T cell proliferation, 3 × 10^5^ splenocytes per well were plated in a 96-well U-bottom plate (Sarstedt) in complete medium, followed by stimulation with 5 μM peptides. As a positive control, cells were incubated with 2.5 μg/mL ConA (Sigma C5275-5MG). After 5 days, cells were centrifuged (2000 rpm, 5 min, 4 °C) and resuspended in RPMI-1640. T cell proliferation was determined using AlamarBlue assay as described above and the percentage of cells, compared to medium-treated cells, was determined.

To evaluate the cytokine production, supernatants obtained after centrifugation were assayed by LEGENDplex Mouse Th Cytokine Panel (BioLegend, San Diego, CA) following the manufacturer’s instructions.

### Statistical Analysis

All values are expressed as mean ± SEM. Data were normally distributed and comparison between groups was performed by one-way ANOVA or two-way ANOVA, followed by Tukey’s post hoc multiple comparison. A value of *p < 0.05 was considered significant.

## Supporting Information Available

Analytical and conformational characterization of epitope peptides and conjugates; additional experimental data for the viability test, antigen-recall assay including the gating strategy and lysosomal degradation study; and experimental details and results of the splenocyte restimulation assay conducted with individual epitope peptides (PDF)
