# Supplementary material for: Design and Characterization of a Multistage Peptide-Based Vaccine Platform to Target Mycobacterium tuberculosis Infection
Source: Bioconjug Chem. 2023 Aug 22;34(10):1738–53. doi: 10.1021/acs.bioconjchem.3c00273 (PMC10587871; doi:10.1021/acs.bioconjchem.3c00273)
Supplement: Supplementary file 1 — bc3c00273_si_001.pdf [file bc3c00273_si_001.pdf]

Supporting Information

Design and characterization of a multistage peptide-based vaccine platform to target *Mycobacterium tuberculosis* infection

Chiara Bellini[1,2]; Emil Vergara[3], Fruzsina Bencs[2,4], Kinga Fodor[5], Szilvia Bősze[6], Denis Krivić[7], Bernadett Bacsa[7], Sára Eszter Surguta[8], József Tóvári[8], Rajko Reljic[3], Kata Horváti[1]*

[1]MTA-TTK Lendület "Momentum" Peptide-Based Vaccines Research Group, Institute of Materials and Environmental Chemistry, Research Centre for Natural Sciences, Budapest, 1117, Hungary;
[2]Hevesy György PhD School of Chemistry, Eötvös Loránd University, Budapest, 1117, Hungary;
[3]Institute for Infection and Immunity, St. George's, University of London, London, SW17 0RE, UK;
[4]Laboratory of Structural Chemistry and Biology, Institute of Chemistry, Eötvös Loránd University, Budapest, 1117, Hungary;
[5]Department of Laboratory Animal Science and Animal Protection, University of Veterinary Medicine, Budapest, 1078, Hungary;
[6]ELKH-ELTE Research Group of Peptide Chemistry, Eötvös Loránd Research Network (ELKH), Eötvös Loránd University, Budapest, 1117, Hungary;
[7]Division of Medical Physics and Biophysics, Gottfried Schatz Research Center, Medical University of Graz, Graz, 8010, Austria;
[8]Department of Experimental Pharmacology and National Tumor Biology Laboratory, National Institute of Oncology, Budapest, 1122, Hungary.

Table S1. Analytical characterization of 5(6)-FAM-labelled peptide epitopes. The fluorescent derivatives of the peptides were obtained by coupling 5(6)-carboxyfluorescein (5(6)-FAM) at the *N*-terminus of the peptides, while the *C*-terminus of all peptides was amidated. The peptides were characterized by analytical RP-HPLC and mass spectrometry.

Epitope name	M_{mo} *Calc.*	M_{mo} *Meas.* [1]	*RT (min)* [2]
**5(6)-FAM GlfT2 (4-12)**	1285.6343	1285.6317	17.6
**5(6)-FAM Ag85B (41-48)**	1228.5553	1228.5522	15.8
**5(6)-FAM CarB (744-754)**	1680.6944	1680.6880	14.9
**5(6)-FAM gap (112-122)**	1451.6834	1451.6759	12.2
**5(6)-FAM CFP10 (32-39)**	1119.4347	1119.4351	14.8
**5(6)-FAM CFP10 (11-25)**	1975.8588	1975.8514	16.0
**5(6)-FAM TB10.4 (20-28)**	1265.5241	1265.5216	15.5
**5(6)-FAM RpfA (377-391)**	2147.0476	2147.0442	15.3
**5(6)-FAM TB8.4 (69-83)**	1991.0054	1991.0019	15.9
**5(6)-FAM mec (2-20)**	2469.3420	2469.3383	16.6
**5(6)-FAM HBHA (185-194)**	1339.6925	1339.6852	11.9
**5(6)-FAM Grol2 (63-78)**	1717.7878	1717.7840	15.1
**5(6)-FAM Mtb32a (309-318)**	1288.5507	1288.5544	14.7
**5(6)-FAM Rv1733c (63-77)**	1954.8234	1954.8148	13.1
**5(6)-FAM PPE15 (1-15)**	2004.8386	2004.8342	14.6
**5(6)-FAM IniB (33-45)**	1705.7988	1705.7986	13.3

[1] M_{mo} Meas. (monoisotopic molecular mass) measured on a Thermo Scientific Q Exactive Focus Hybrid Quadrupole-Orbitrap Mass Spectrometer. [2] Retention time on Phenomenex Jupiter C12, gradient: 5%–100% B, 20 min. According to the HPLC analysis, the purity of the peptides was always above 95%.

Figure S1-S4. Chemical characterization of CFP10 (32-39), GlfT2 (4-12), HBHA (185-194), and PPE15 (1-15). The retention time was obtained on a Phenomenex Jupiter C12 column with the applied linear gradient. The peak detection was measured at λ = 220 nm. The MS spectrum was measured on a Thermo Scientific Q Exactive Focus Hybrid Quadrupole-Orbitrap Mass Spectrometer. Detailed instrument and method parameters were described in the methods section.

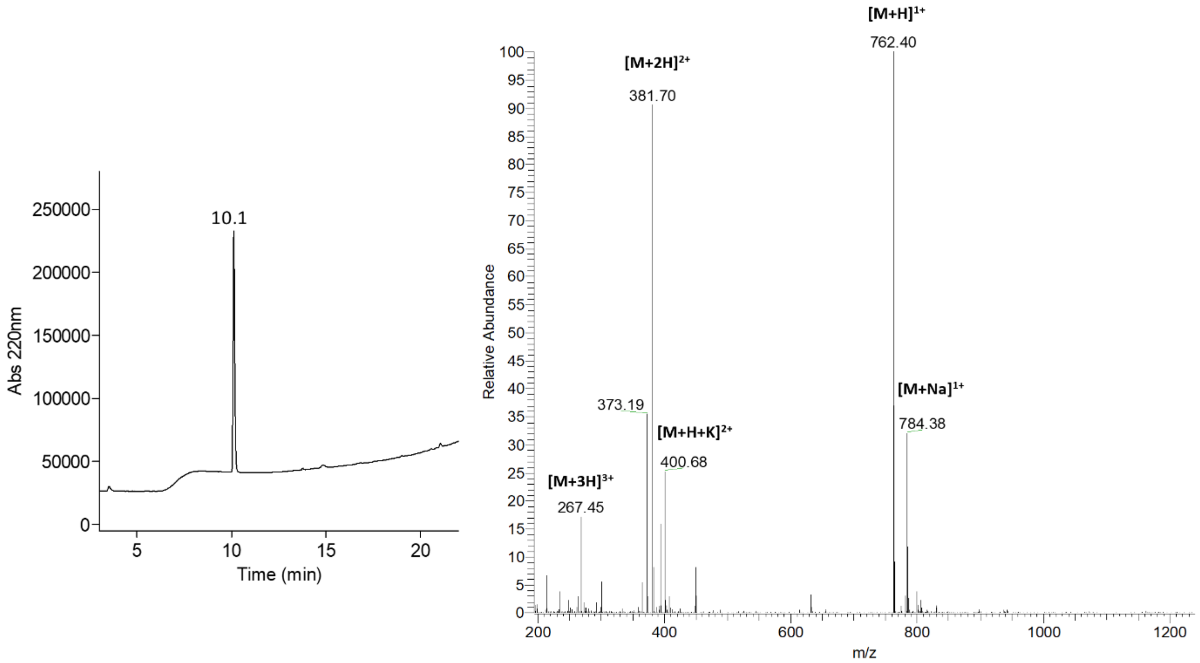

Figure S1. RP-HPLC chromatogram and ESI-HRMS spectrum of **CFP10 (32-39)**.

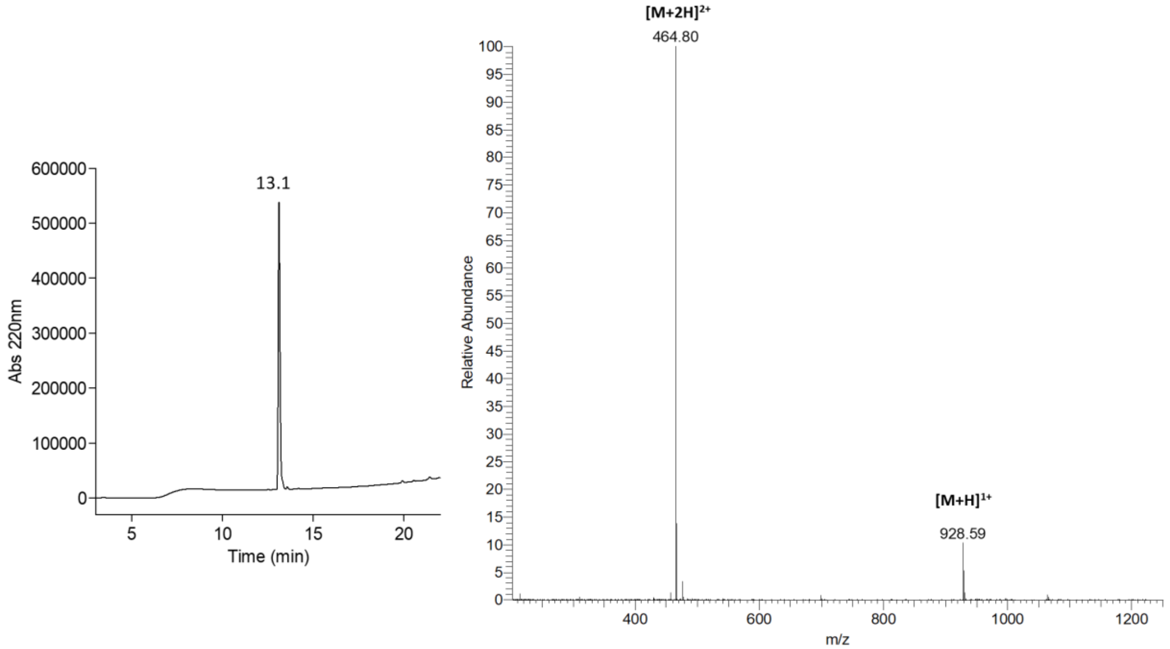

Figure S2. RP-HPLC chromatogram and ESI-HRMS spectrum of **GlfT2 (4-12)**.

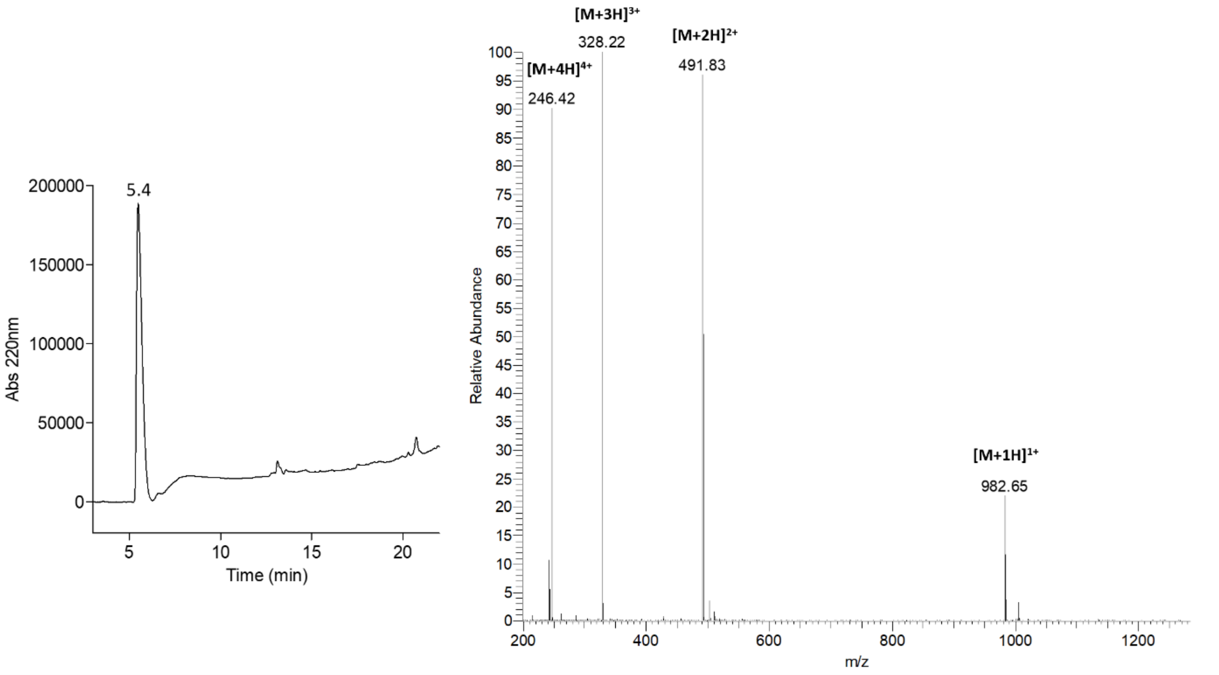

Figure S3. RP-HPLC chromatogram and ESI-HRMS spectrum of **HBHA (185-194)**.

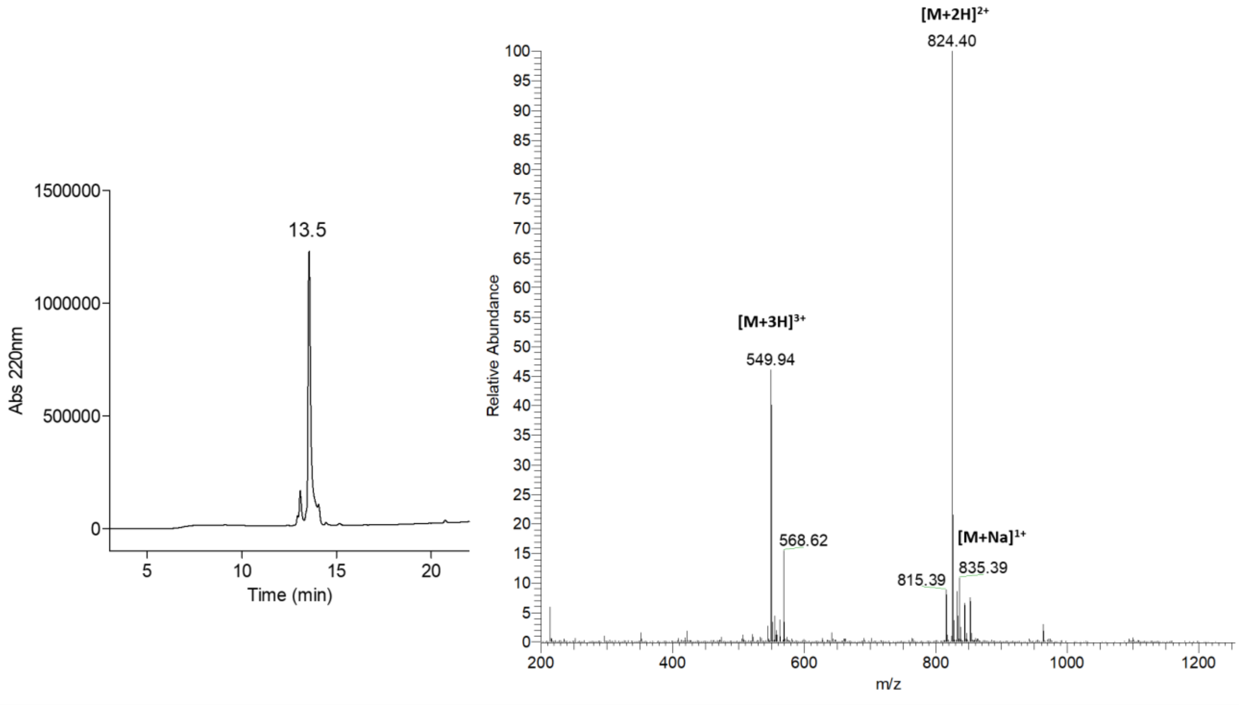

Figure S4. RP-HPLC chromatogram and ESI-HRMS spectrum of **PPE15 (1-15)**.

Figure S5. AlamarBlue viability assay on MonoMac6 cell line.

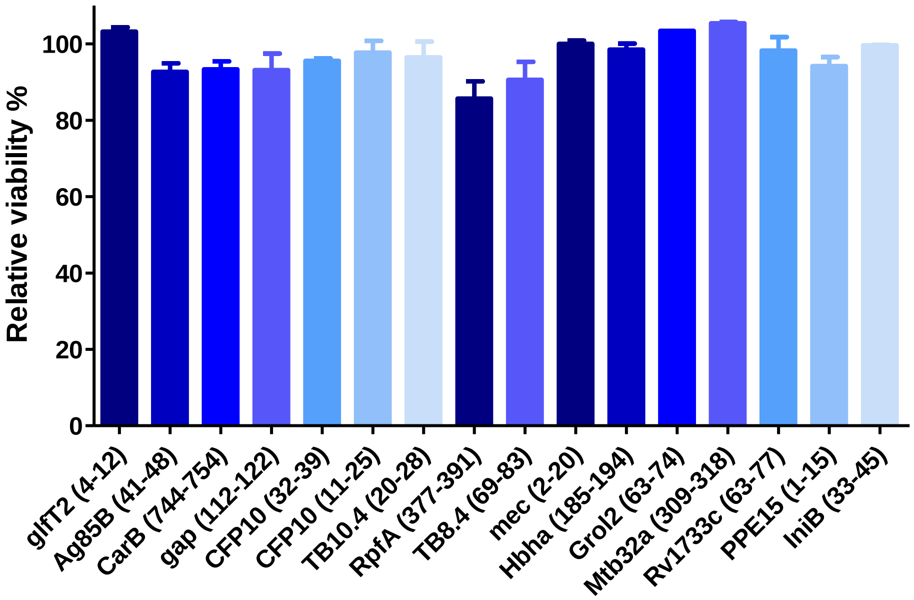

Figure S5. The relative viability values (Relative viability %) were obtained by comparing the viability of cells treated with the peptides (100 µM) to the untreated cells, after 24 hours of incubation. Each bar represents a mean value of four parallel measurements ± SEM. None of the peptides showed significant toxicity compared to the untreated cells ($p > 0.05$).

Figure S6. Antigen recall assay on human PBMCs from *Mtb*-sensitized donors.

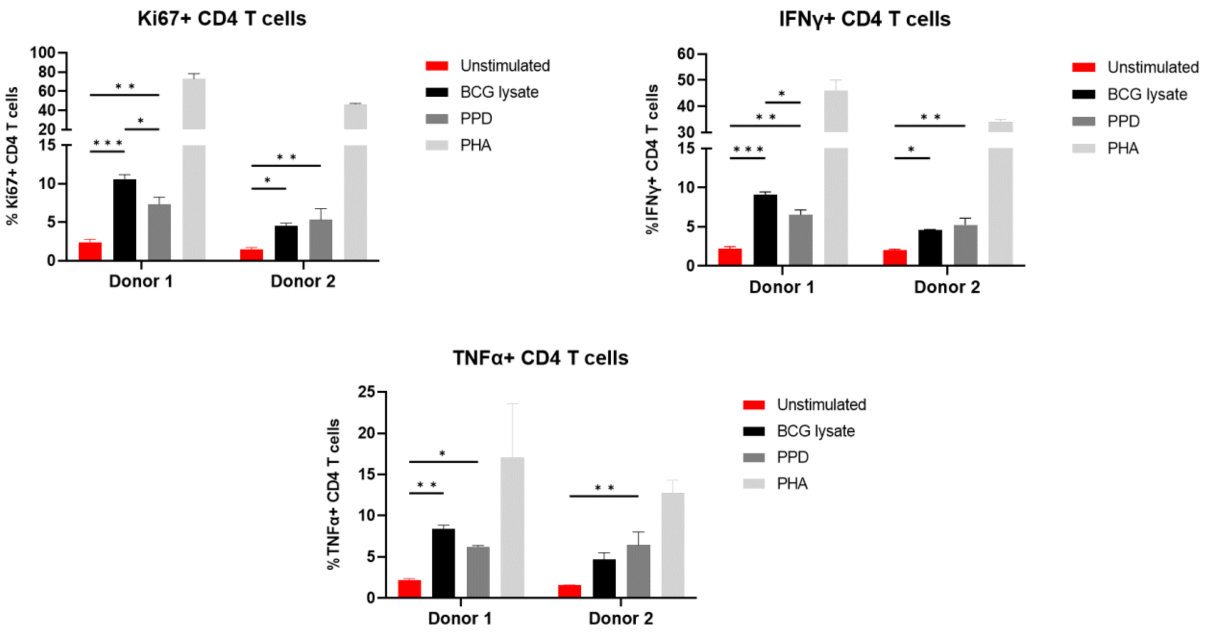

Figure S6. Profiling of the T cell activation in human Mtb-sensitized PBMCs. Cells were spiked with either *Mtb* PPD (5 µg/mL), M. bovis BCG whole cell lysate (5 µg/mL), or PHA (5 µg/mL) for 5 days and then analyzed by flow cytometry, probing for intracellular

Th1 cytokines (IFNγ and TNFα) and for the proliferation marker Ki67. Statistical analysis was performed using two-way ANOVA followed by post-hoc Tukey's test, *p ‹0.05, **p ‹0.01, ***p ‹0.001.

Gating strategy.

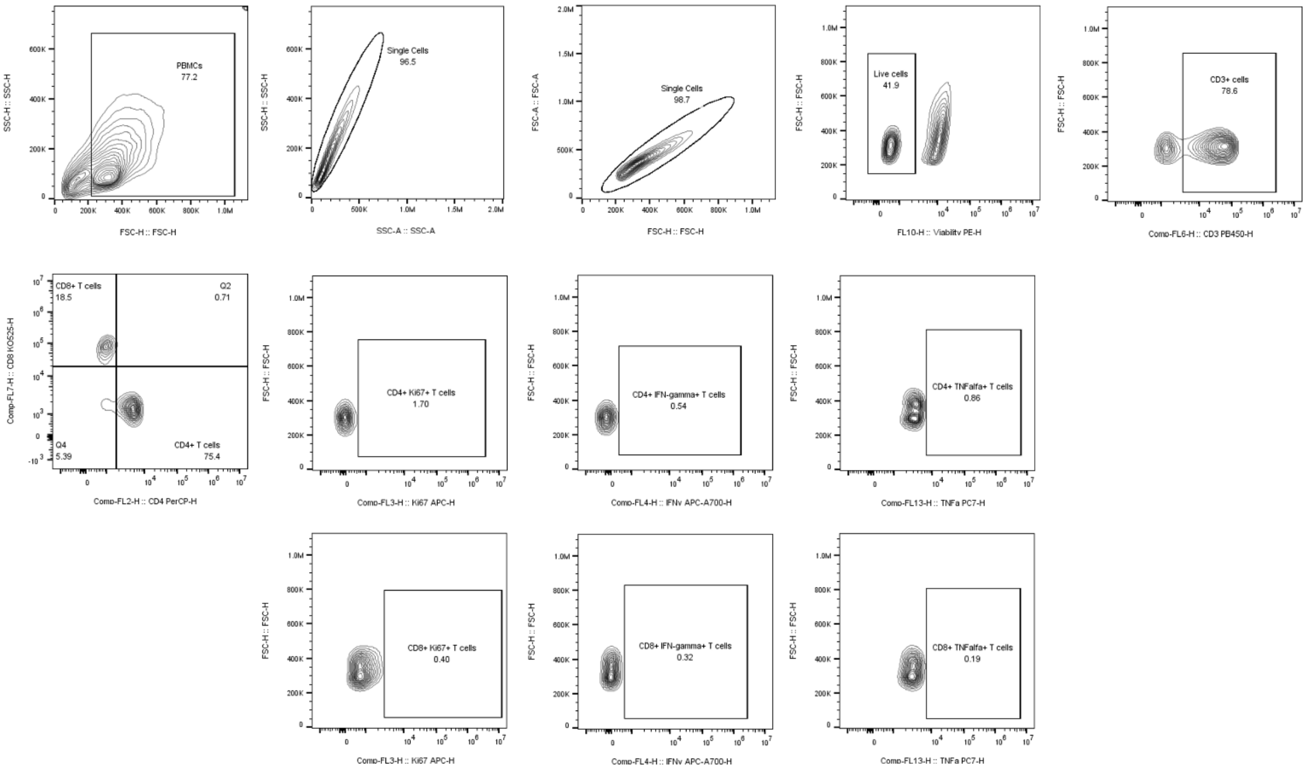

Table S2. Analytical characterization of the multi-epitope branched conjugates. Analytical characterization of the multi-epitope conjugates. The fluorescent derivatives of the conjugates were obtained by coupling 5(6)-carboxyfluorescein (5(6)-FAM) at the *N*-terminus of the cysteine-elongated PPE15 (1-15) peptide.

	M_{mo} calculated	M_{mo} meas. [1]	RT (min) [2]
**Ac-CGHP**	4621.4547	4621.4550	13.0
**Pal-CGHP**	4817.6938	4817.6520	13.1*
**Ac-CGHP- *Cf***	4979.5024	4978.4660	15.2
**Pal-CGHP- *Cf***	5175.7415	5175.6980	18.1

[1] M_{mo} Meas. (monoisotopic molecular mass) measured on a Thermo Scientific Q Exactive Focus Hybrid Quadrupole-Orbitrap Mass Spectrometer. [2] Retention time on Phenomenex Jupiter C12, gradient: 5%–100% B, 20 min (*gradient: 40-100% B). According to the HPLC analysis, the purity of the conjugates was above 95%.

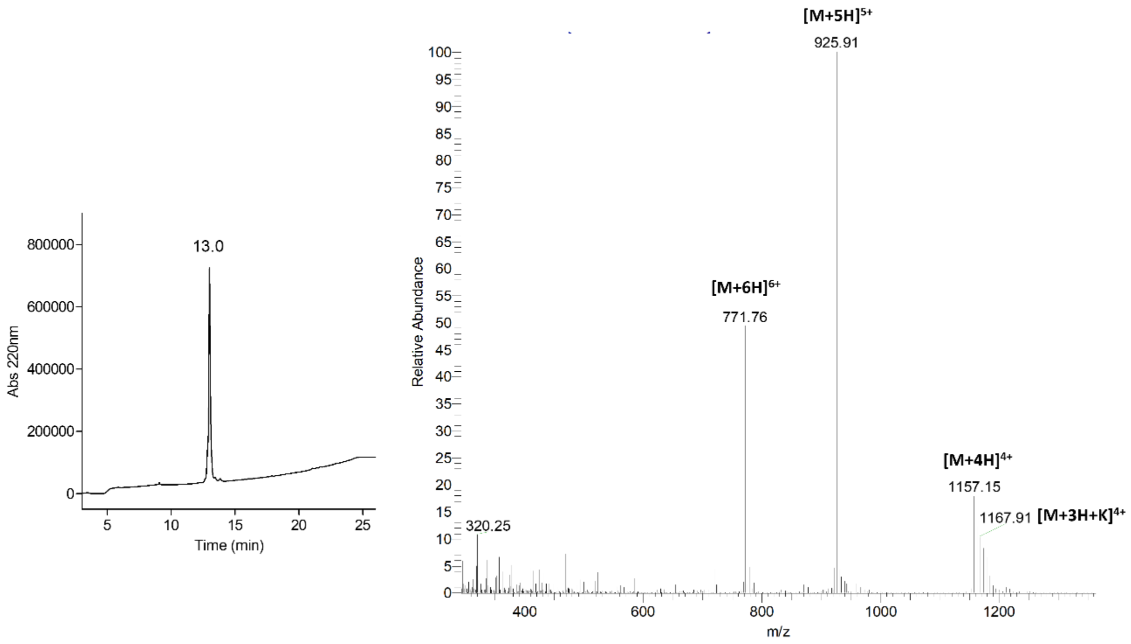

Figure S7. RP-HPLC chromatogram and ESI-HRMS spectrum of **Ac-CGHP**.

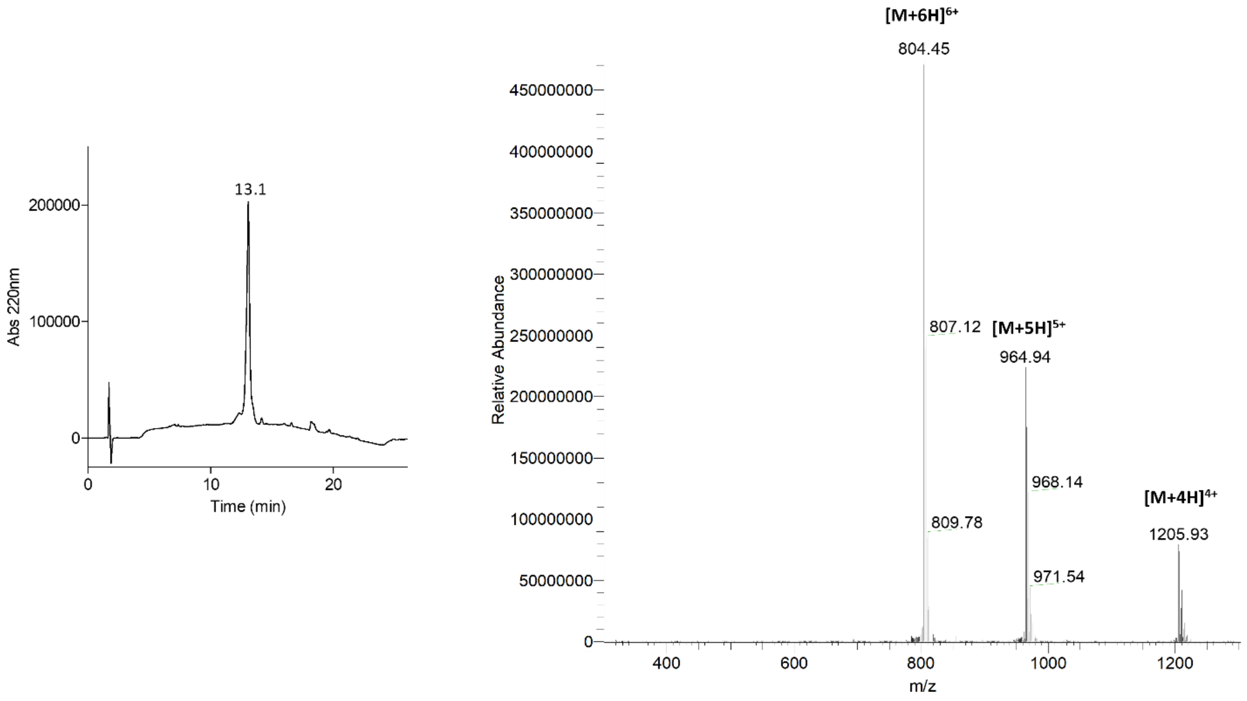

Figure S8. RP-HPLC chromatogram and ESI-HRMS spectrum of **Pal-CGHP**.

Conformation study. In Figure **S9**, ECD spectra of the epitope peptides at various TFE ratios are presented. CFP10 (**A**) and HBHA (**C**) remained unstructured: CFP10 is too short to form turns while the polar sidechains of HBHA bear a charge at pH = 7.4, impeding the formation of a secondary structure. The GlfT2 (**B**) and PPE15 (**D**) peptides showed a propensity to fold and form a turn at higher TFE concentrations. This could be explained by the effect of TFE on the intrinsic properties of the two peptides, namely the relatively apolar sidechain content of GlfT2 and the PP motif of PPE15.

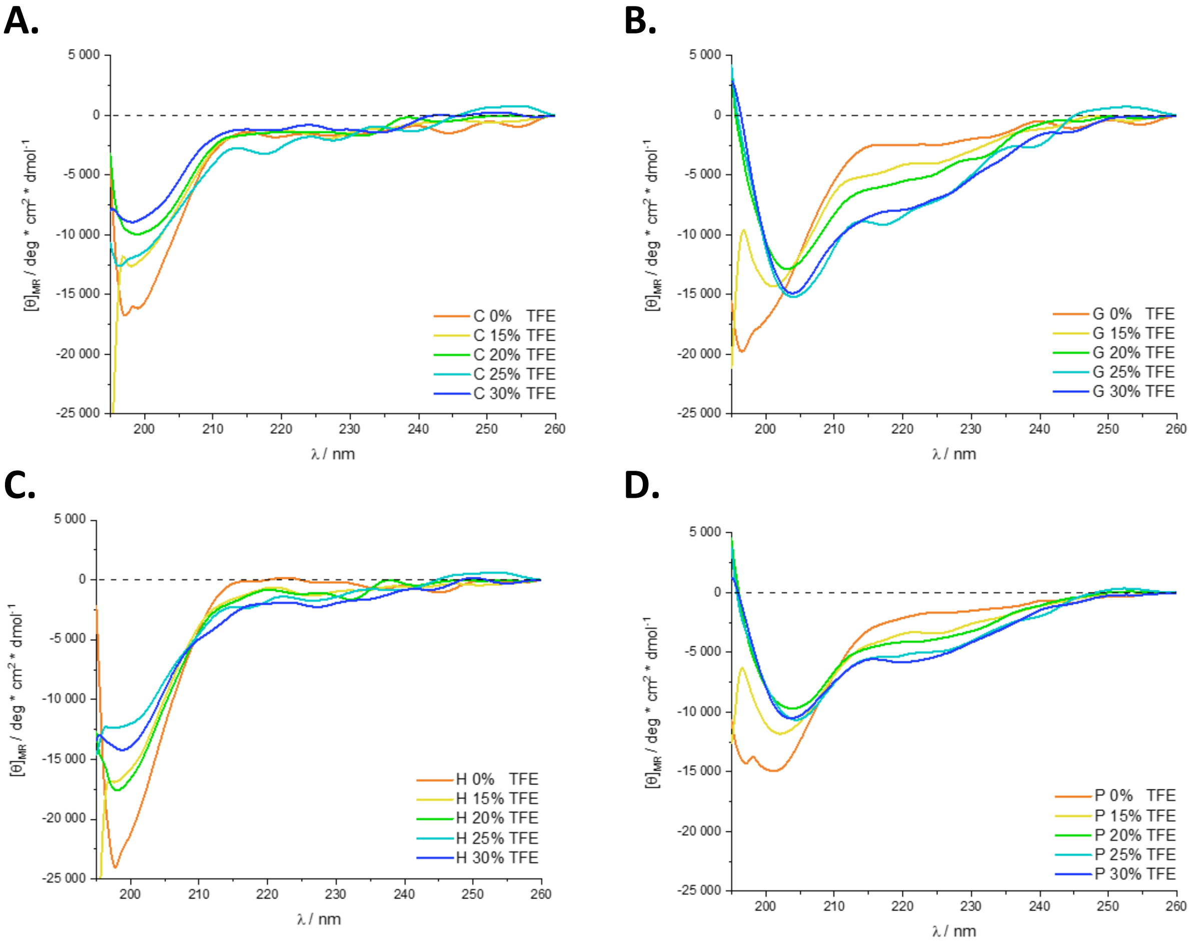

Figure S9. ECD spectra of epitope peptides at different TFE ratios: **(A)** CFP10, **(B)** GlfT2, **(C)** HBHA, **(D)** PPE15. The measurements were conducted with the following parameter: peptide concentration was 26 µM, the solvent was PBS buffer containing different % of TFE, pH= 7.4, T= 25°C.

Figure **S10** shows the ECD spectra of conjugates (**Ac-CGHP** and **Pal-CGHP**) and the 1:1:1:1 epitope peptide mixture recorded at different TFE concentrations. The spectra of **Ac-CGHP** and the peptide mixture were recorded at a TFE concentration range of 0-40% TFE, while 30% of TFE was required to dissolve the palmitoylated conjugate because of its poor solubility in an aqueous solution. **Ac-CGHP** has a dynamic, unordered structure in PBS, whereas both conjugates tend to fold and form a turn or helical secondary structure in a less hydrophilic environment (**A** and **B**, blue line). Epitope peptides do not affect each other's secondary structure (**C**).

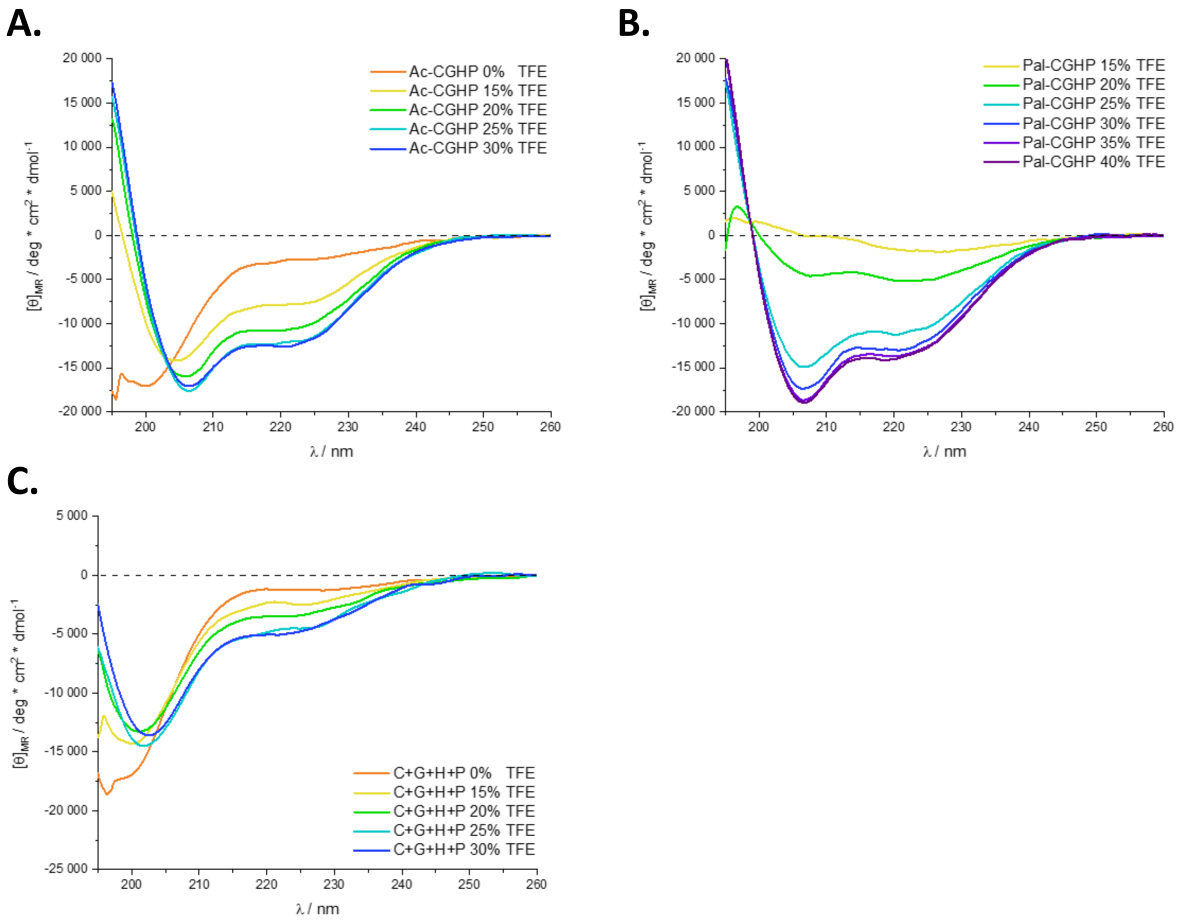

Figure S10. ECD spectra of conjugates and mix of epitope peptides: **Ac-CGHP (A)**, **Pal-CGHP (B)**, and the 1:1:1:1 epitope mixture **(C)**. The measurements were conducted with the following parameters: concentration= 26 µM, solvent = PBS buffer, pH = 7.4, T = 25°C.

Degradation study of Ac-CGHP in the rat liver lysosomal homogenate.

	RT (min)	M_{mo} calculated	Identified peptide fragments
**(1)**	**7.88**	4621.4464	**Ac-CGHP**
**(2)**	**2.68**	2729.52630	(**Ac-CGHP**) - MDFG
**(3)**	**3.86**	468.1672	MDFG
**(4)**	**5.44**	402.2469<br>2796.4186<br>3179.6816	ASLL<br>APAK((mal)PPE15-Cys)KAAAK*<br>RVKAPAK((mal)PPE15Cys)KAAAK*
**(5)**	**6.49**	804.3852	VESTAGSL
**(6)**	**8.87**	917.469	VESTAGSLL

Table S3. Peptide fragments obtained by the degradation of **Ac-CGHP** in rat liver lysosomal homogenate. Only fragments providing clearly visible peaks are reported. *(mal)PPE15-Cys) indicates the maleimide group + the cysteine-elongated PPE15 (1-15) peptide.

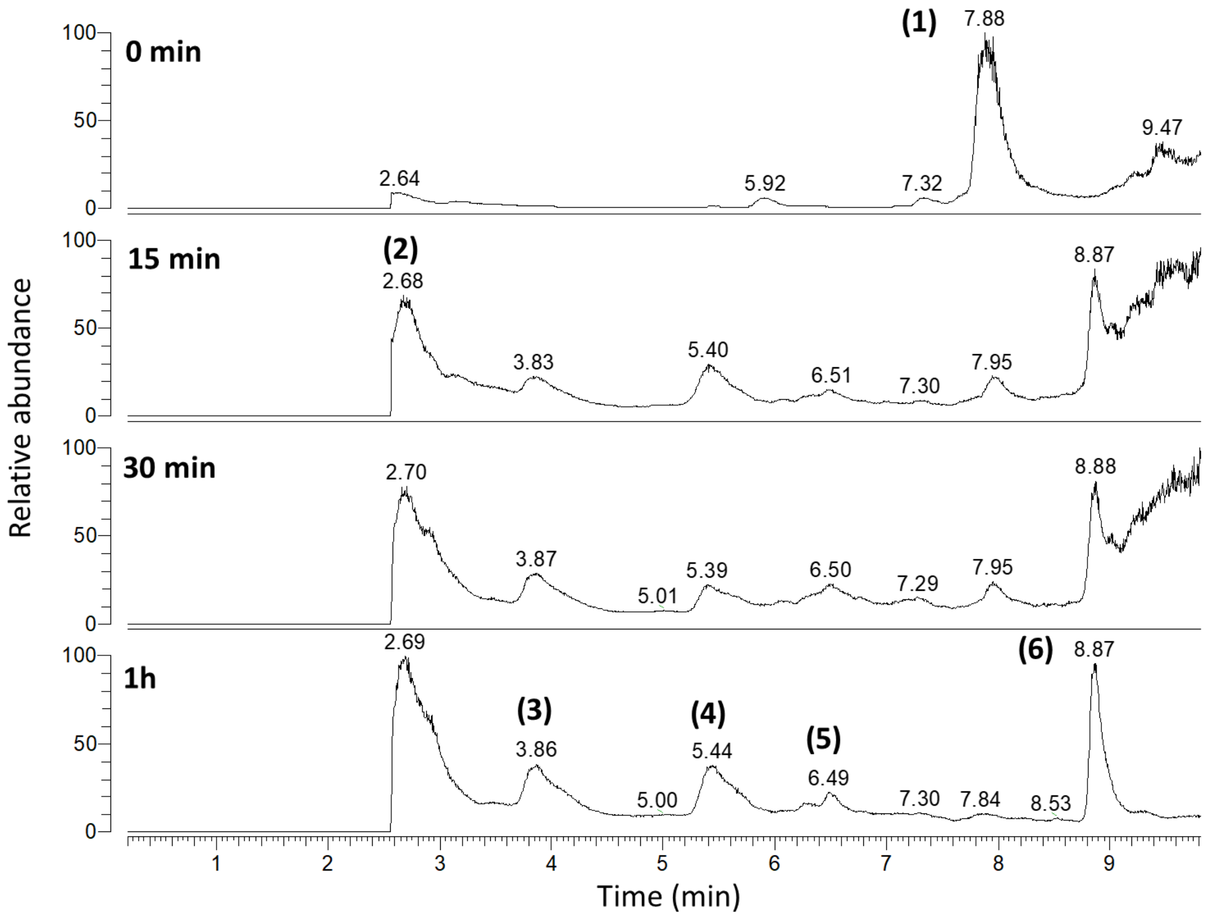

Figure S11. Mass Spectrometry Extracted Ion Chromatograms (EIC) at the collection time points (0 min, 15 min, 30 min, 1 h).

Restimulation assay with individual epitope peptides. Epitope-specificity of **Pal-CGHP** immunization was assessed by a restimulation assay on splenocytes from immunized mice. Frozen splenocytes of mice vaccinated with **Pal-CGHP** and untreated control mice were thawed in 10% FBS-containing RPMI media. After washing steps, cell viability and membrane integrity were determined using trypan blue staining. Then, cells were seeded in a 96-well round bottom plate (300.000 cells / 100 µL media), and ndividual epitope peptides were added to the cells in 100 µL media at 10 µM final concentration. RPMI media and ConA (1.25 µg/mL) were used as negative and positive controls, respectively. After 5 days of incubation, plates were centrifuged, and 100 uL of the supernatant was replaced with 10% AlamarBlue solution in RPMI. Following a 4-hour incubation, the fluorescence was detected at λ_{Em} = 610/10 nm (λ_{Ex} = 530/30) using a Synergy H4 reader (BioTek, Winooski, VT). All measurements were performed in triplicates, and the percentage of proliferation, compared to medium-treated cells, is presented.

Data proved (Figure **S12**), that the response of **pal-CGHP** immunized mice to the epitope sequences is specific, which suggests that the used conjugation method did not compromise the epitope recognition and peptide antigenicity.

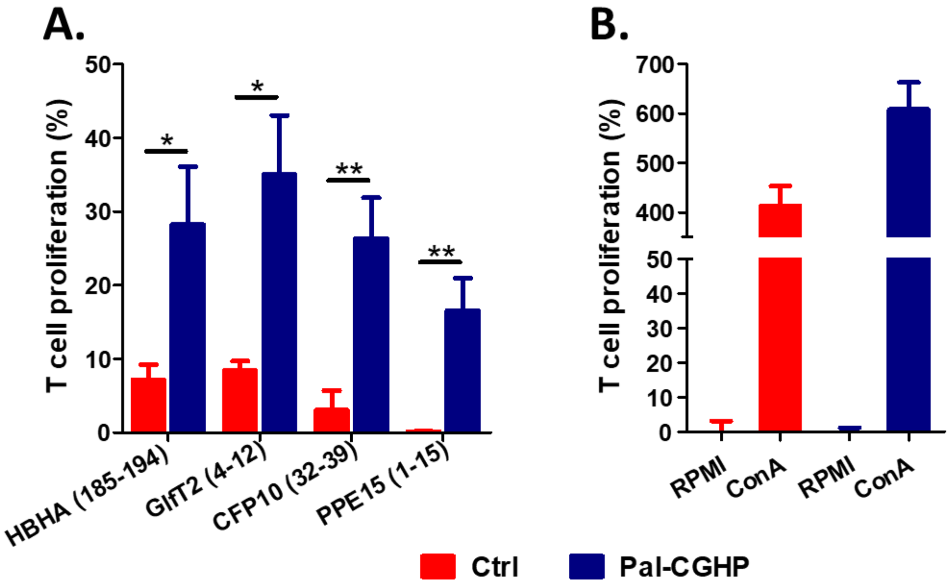

Figure S12. Peptide specificity of the **pal-CGHP** induced immune response. Spelnocytes from control mice and **pal-CGHP** conjugate-immunized mice were re-stimulated with individual epitope peptides (**A**). Panel **B** shows the relevant assay controls such as RPMI medium treatment and ConA aspecific stimulant. Represented data are mean ± SEM. (n = 3). Statistical analysis was performed using one-way ANOVA followed by Tukey's post-hoc test. Statistical significance: *p ‹0.05, **p ‹0.01.
